# Pharmacokinetic, Tissue Distribution, Metabolite, and Toxicity Evaluation of the Matrine Derivative, (6aS, 10S, 11aR, 11bR, 11cS)-10-Methylaminododecahydro-3a, 7a-Diaza-benzo (de) Anthracene-8-thione

**DOI:** 10.3390/molecules29020297

**Published:** 2024-01-06

**Authors:** Liuyan Li, Fangfang Lu, Shuqin Ding, Xiaoying Wang, Weibiao Wang, Wannian Zhang, Weiheng Xu, Chunlin Zhuang, Zhenyuan Miao, Xueqin Ma

**Affiliations:** 1 Department of Pharmaceutical Analysis, School of Pharmacy, Key Laboratory of Protection, Development, and Utilization of Medicinal Resources in Liupanshan Area, Ministry of Education, Ningxia Medical University, 1160 Shenli Street, Yinchuan 750004, China; liliuyan1219@126.com (L.L.); lffang55@163.com (F.L.); md-dshn@163.com (S.D.); wangxy_0816@163.com (X.W.); 18973888070@163.com (W.W.); 18616371838@163.com (W.Z.); zclnathan@163.com (C.Z.); 2 Department of Pharmacy, Ningxia Health Vocational and Technical College, Intersection of Starlight Avenue and Xueyuan Road, Shizuishan 753000, China; 3 School of Pharmacy, Second Military Medical University, 325 Guohe Road, Shanghai 200433, China; xuweiheng7114@163.com

**Keywords:** MASM, pharmacokinetics, tissue distribution, metabolites, toxicity evaluation

## Abstract

MASM, a structurally modified derivative of matrine, exhibits superior efficacy in reducing inflammation and liver injury in rats when compared to matrine. This study aims to investigate the pharmacokinetic profile and acute toxicity of MASM. Pharmacokinetic results revealed that MASM exhibited rapid absorption, with a *T_max_* ranging from 0.21 ± 0.04 h to 1.31 ± 0.53 h, and was eliminated slowly, with a *t*_1/2_ of approximately 10 h regardless of the route of administration (intravenous, intraperitoneal, or intragastric). The absolute intragastric bioavailability of MASM in rats was determined to be 44.50%, which was significantly higher than that of matrine (18.5%). MASM was detected in all rat tissues including the brain, and through the utilization of stable isotope-labeled compounds and standard references, ten metabolites of MASM, namely sophocarpine, oxysophocarpine, and oxymatrine, were tentatively identified. The LD_50_ of MASM in mice was determined to be 94.25 mg/kg, surpassing that of matrine (83.21 mg/kg) based on acute toxicity results. Histopathological and biochemical analysis indicated no significant alterations in the primary organs of the low- to medium-dosage groups of MASM. These findings provide valuable insights into the efficacy and toxicity profile of MASM.

## 1. Introduction

Inflammation has been identified as the seventh hallmark of malignant tumors, and the immune microenvironment resulting from chronic inflammation serves as the breeding ground for tumorigenesis. Liver cancer exemplifies this mechanism prominently. China is recognized as a region with a high prevalence of viral hepatitis, and the elevated incidence and mortality rates of liver cancer are intricately linked to the heightened occurrence of hepatitis. Fibrosis plays a pivotal role in connecting inflammation with liver cancer, as most cases of hepatocellular carcinoma are accompanied by varying degrees of cirrhosis and liver fibrosis [1]. Currently, there is no effective method to counteract the advancement of liver fibrosis, thereby impeding significant advancements in the prevention and management of liver cancer. Moreover, liver cancer has witnessed a rise in its ranking from the third to the second highest fatality rate among cancer types between 2018 and 2020 [2]. Until now, surgical resection remains the foremost treatment modality for liver cancer, representing the sole potentially curative option for individuals diagnosed with early-stage disease.

**Figure 1:**
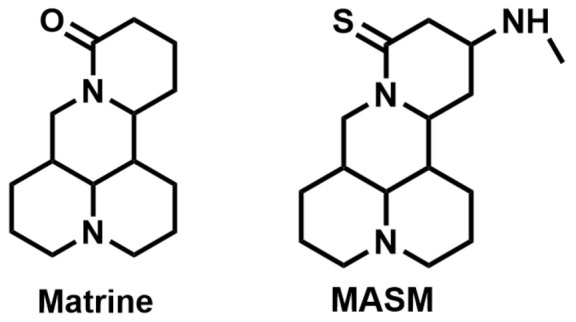
The structures of Matrine and MASM.

Regrettably, a significant number of liver cancer patients are frequently diagnosed during the intermediate and advanced stages of the disease, thereby missing the optimal window for treatment [3]. Given that the development of liver cancer primarily arises from chronic hepatitis and cirrhosis, blocking the progression of hepatitis into cancer has emerged as a crucial approach to preventing the onset of liver cancer. Matrine (MT), a natural alkaloid derived from the traditional Chinese herbs, *Sophora flavescentis* and *Sophora alopecuroides*, has demonstrated remarkable anti-inflammatory properties and the potential to hinder fibrogenesis in the liver [4]. However, it has also been observed that matrine exhibits significant neurotoxicity and developmental toxicity, as well as low bioavailability [5]. Consequently, structural modification represents an effective strategy for mitigating the associated toxicity. More and more articles were reported to study the structural modification of MT to find better compounds for clinical selection [6]. A novel derivative of matrine, namely (6aS, 10S, 11aR, 11bR, 11cS)-10-methylaminododecahydro-3a, 7a-diaza-benzo (de) anthracene-8-thione (MASM), has demonstrated a wide range of pharmacological activities, including anti-fibrosis, anti-tumor, and immune regulation properties [7]; the structures of MASM and matrine are presented in Figure 1. However, there is a scarcity of studies investigating the pharmacokinetics and toxicity of MASM in vivo. Thus, in our present study, the investigation of the bioavailability, tissue distribution, metabolites, and acute toxicity of MASA in vivo was conducted through the utilization of a pharmacokinetic study in rats and acute toxicity in mice.

## 2. Results

### 2.1. Blood Concentration–Time Profile of MASM

#### 2.1.1. Selection of Dose

Sprague-Dawley rats were administered different doses of MASM based on the routes of administration, encompassing intravenous, intraperitoneal, and intragastrical administration. Given that matrine injection has been clinically used in China at a dose of 0.15 g, and MASM is a derivative of matrine, the dose of MASM for rats via intravenous administration was set at 15 mg/kg (dissolved in saline) according to the conversion of the human clinical dose. Subsequently, for intraperitoneal and intragastric administration, the dosages were determined based on the LC-MRM-MS/MS sensitivity. Consequently, dosages of 80 mg/kg (suspended in saline) for intraperitoneal and intragastric 60 mg/kg (dissolved in saline) administration were selected.

#### 2.1.2. Method Validation

Supplementary Figure S1 displays the extracted ion chromatograms (EICs) of the internal standard (IS) and MASM. The absence of any potential endogenous interferences at the retention times of both MASM and IS suggests that the specificity of the method was acceptable. The regression equations for MASM were determined through weighted linear least squares regression. Additionally, the results of specificity, linearity, precision, accuracy, matrix effect, extraction recovery, and stability all complied with the requirements, which further supported the reliability and accuracy of this method. All the data can be found in Supplementary File S2.

#### 2.1.3. Blood Concentration–Time Profiles of MASM

**Figure 2:**
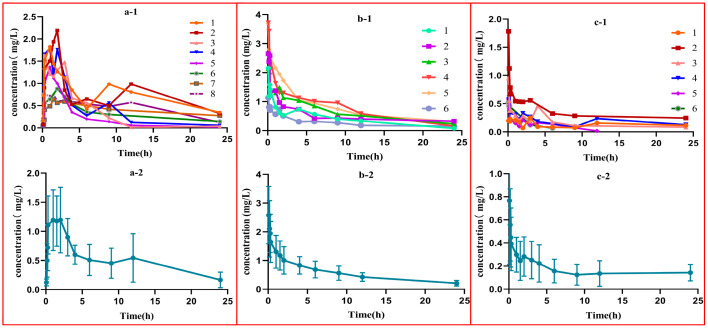
The plasma concentration–time curves of MASM in rats. (**a-1**,**a-2**): each rat and the mean plasma concentration–time curves of MASA via intragastrical administration at a dose of 60 mg/kg; (**b-1**,**b-2**): each rat and the mean plasma concentration–time curves of MASA via intraperitoneal administration at a dose of 80 mg/kg; (**c-1**,**c-2**): each rat and the mean plasma concentration–time curves of MASA via intravenous administration at a dose of 15 mg/kg.

The plasma concentration–time profiles of MASM in rats are illustrated in Figure 2 via three distinct routes. The primary pharmacokinetic parameters of MASM were calculated and summarized using non-compartmental analysis, as outlined in Table 1. Expectedly, MASM was rapidly detected in the blood following intravenous and intraperitoneal administration, and when administered intragastrically, the plasma concentration of MASM displayed a gradual increase, reaching its maximum concentration (*C_max_*) at 1.3 h, followed by a gradual decline. The elimination half-life of MASM following intravenous, intraperitoneal, and intragastrical administration was determined to be 10.51 ± 8.84 h, 9.97 ± 3.95 h, and 10.25 ± 4.95 h, respectively. These results demonstrated that MASM exhibited rapid absorption and slow elimination. Additionally, the bioavailability of MASM after intragastrical administration was determined to be 44.50%, which was significantly higher than that of matrine (reported to have a bioavailability of 18.5%) [5].

### 2.2. Tissue Distribution of MASM

#### 2.2.1. Method Validation

Supplementary Figure S2 depicts the EIC of IS and MASM in various tissues of rats. Similar to the experiment on blood concentration–time, the determination of MASM or IS was not influenced by any potential endogenous interferences. The calibration curves of MASM in each blank tissue matrix are presented in Supplementary File S3, demonstrating a strong linear relationship within the range of 5 ng/mL to 1000 ng/mL.

#### 2.2.2. Tissue Distribution

**Figure 3:**
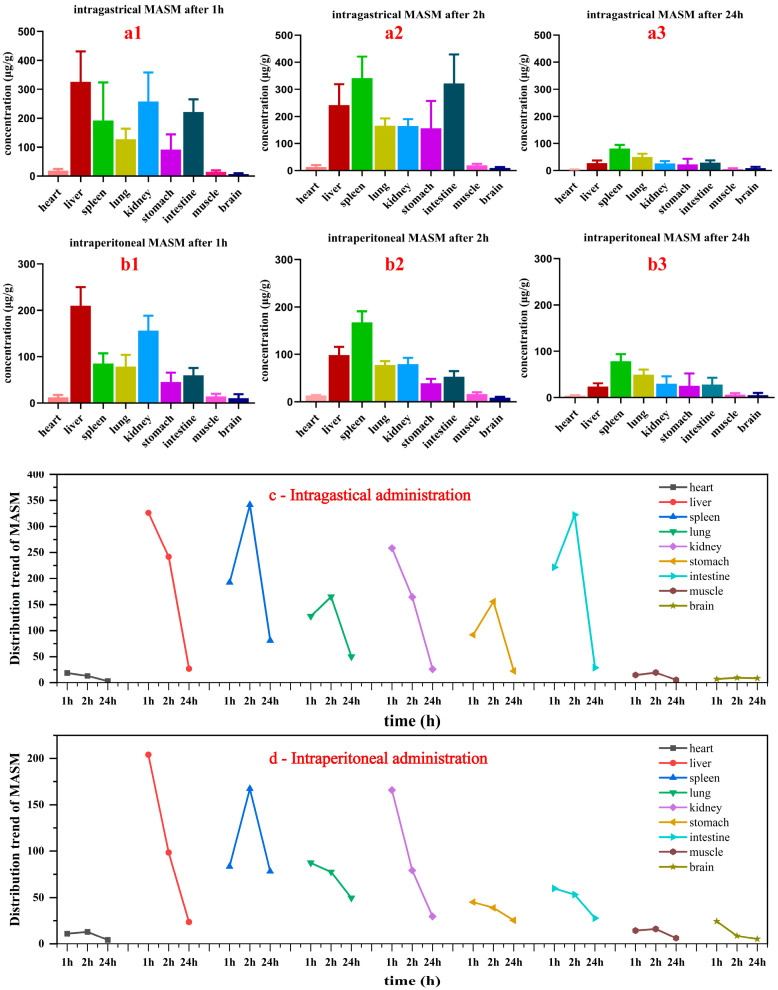
Tissue distribution of MASM at different times in rats. (**a1**–**a3**): tissue distribution of MASM at 1, 2, 24 h after intragastric administration, respectively; (**b1**–**b3**): tissue distribution of MASM at 1, 2, 24 h after intraperitoneal injection, respectively; (**c**,**d**) distribution trends of MASM in different tissues at different time points after intragastric and intraperitoneal injection, respectively.

To evaluate the uptake and distribution of MASM in major tissues of rats, tissue distributions were measured at 1 h, 2 h, and 24 h after intragastrical and intraperitoneal administration, respectively. As illustrated in Figure 3, the *C_max_* of MASM in liver tissue was achieved within 1 h after intragastrical and intraperitoneal administration. At 2 h and 24 h, the spleen exhibited higher concentrations of MASM compared to other organs. This indicated that regardless of the route of administration (intragastric or intraperitoneal), MASM is primarily distributed in the liver, kidney, and spleen tissues, all of which have a sufficient blood supply. These three organs were also speculated to be the main metabolic organs of MASM. The lung, stomach, and intestine also showed MASM distribution, albeit to a lesser extent. These findings provided a pharmacokinetic basis for the potential therapeutic use of MASM in liver fibrosis, hepatitis, liver cancer, and other liver tissue diseases.

### 2.3. Metabolite Profiling of MASM

**Figure 4:**
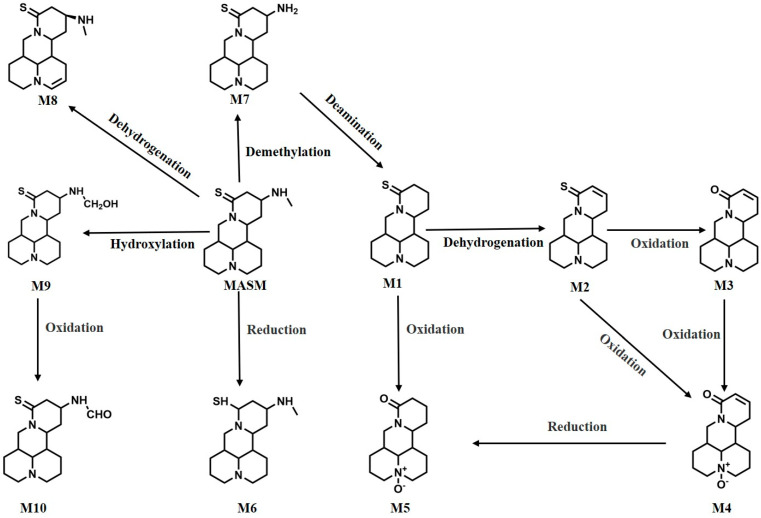
Possible metabolic pathways of MASM in rats.

**Figure 5:**
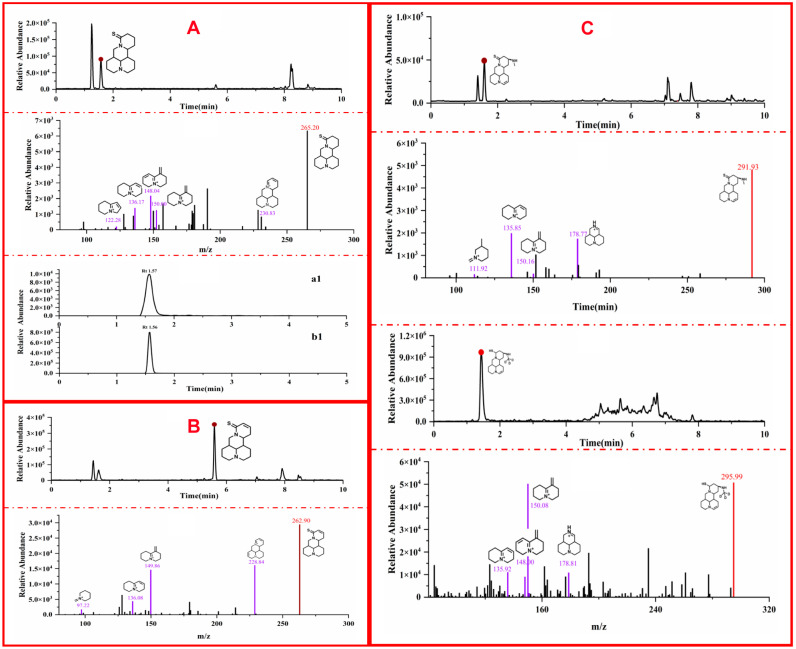
EIC and MS/MS spectra of different metabolites. (**A**) represents the EIC, MS/MS spectrum of M1 as well as the EIC of standard reference where a1 is the sample and b1 is the standard reference; (**B**) demonstrates the EIC and MS/MS spectrum of M2; (**C**) illustrates the EIC and MS/MS spectra of M8 and DM8, respectively.

The identification of the in vivo metabolites of MASM was conducted using UHPLC-QQQ-MS/MS combined with standard references and deuterium-labeled MASM. The findings are presented in Table 2, while the proposed primary metabolic pathway of MASM in rats is depicted in Figure 4. To provide a comprehensive understanding of the metabolite identification process, three representative metabolites of MASM are presented, with their identification relying on primary and secondary mass spectrometry data, standard references, and deuterium-labeled MASM. The remaining metabolites are detailed in Supplementary File S4. For instance, the identification of the metabolite, M1, as a demethylation product of MASM was accomplished through the utilization of MS, MS/MS data, and the standard reference. The comprehensive identification process involved determining the molecular formula of metabolite, M1, as C_15_H_24_N_2_S (*m*/*z* 265.17 [M+H]^+^), which exhibited a mass 29 Da lower than that of MASM (*m*/*z* 294.19 [M+H]^+^). The major MS/MS fragment ions of M1, as indicated by the characteristic fragment ions observed in the MS/MS spectrum, were observed at *m*/*z* 230.83, 150.00, 136.17, 122.28, and 148.04. Moreover, the identification of M1 as the demethylation metabolite of MASM was accomplished using the standard reference synthesized in our laboratory. Figure 5A displays the EIC diagram, MS/MS spectrum, and EIC of the standard reference. Secondly, the metabolite, M2, of MASM was identified as the dehydrogenation product of MASM through the utilization of MS and MS/MS data. The detailed processes of identification are described as follows: the molecular formula of metabolite, M2, was deduced as C_15_H_22_N_2_S (*m*/*z* 263.15 [M+H]^+^), exhibiting a mass of 2 Da lower than that of M1 (*m*/*z* 294.19 [M+H]^+^). The major MS/MS fragment ions of M2 were observed at *m*/*z* 228.84, 149.86, 136.08, and 97.22, demonstrating a similarity to the MS cleavage behavior of M1. Consequently, M2 was identified as a dehydrogenation metabolite of M1. The EIC diagram and MS/MS spectrum are shown in Figure 5B. Thirdly, through the utilization of MS, MS/MS data, and deuterium-labeled MASM, the metabolite, M8, of MASM was determined to be a dehydrogenation product of MASM. The evaluation processes were conducted as follows: the molecular formula of metabolite, M8, was established as C_16_H_25_N_3_S (*m*/*z* 292.18 [M+H]^+^), exhibiting a mass of 2 Da lower than MASM (*m*/*z* 294.19 [M+H]^+^). The major MS/MS fragment ions of M8 were found at *m*/*z* 111.92, 178.77, 150.16, and 135.85, which aligned with the characteristic cleavage pattern of matrine. Additionally, our lab synthesized deuterium-labeled MASM to further support our proposed structure. The major MS/MS fragment ions of the deuterium-labeled MASM were observed at *m*/*z* 178.81, 150.08, and 135.92, which closely resembled the fragments of M8. Consequently, M8 was identified as a dehydrogenation metabolite of MASM. The EIC, MS/MS spectrum of M8 and deuterium-labeled MASM are all depicted in Figure 5C.

### 2.4. Acute Toxicity of MASM

#### 2.4.1. LD_50_ of MASM on Mice via Intravenous Administration

**Figure 6:**
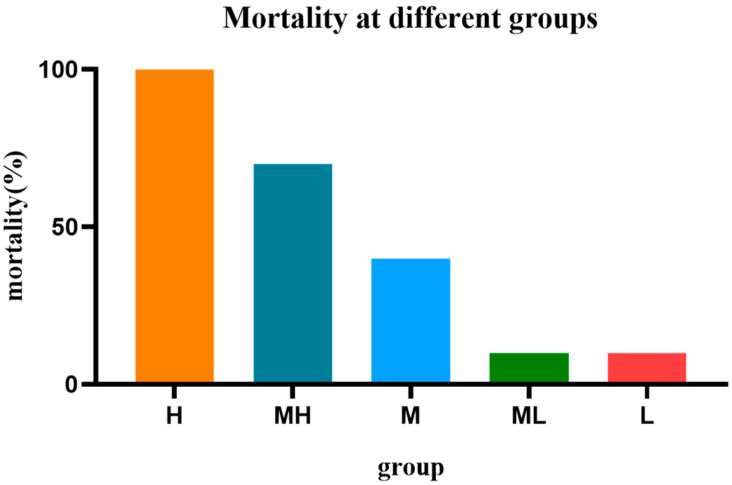
The mortality rate of mice treated with different doses of MASM after intravenous administration. The experimental group was divided into six groups according to the different doses. N represents the control group; H represents the high group; MH represents the medium-high group; M represents the medium group; ML represents the medium-low group; L represents the low group.

The mortality rates of various dosage groups of MASM administered intravenously were compared to those of the control group, as depicted in Figure 6. Upon death, the mice exhibited characteristic symptoms such as convulsions, increased salivary secretion, urination, and defecation. Conversely, the non-deceased mice displayed normal indexes. The LD_50_ of MASM administered intravenously was determined to be 94.25 mg/kg, with a 95% confidence interval ranging from 70.83 to 129.01 mg/kg.

#### 2.4.2. Effect of MASM on the Body Weight, Tissue, and Organ Morphology of Mice

**Figure 7:**
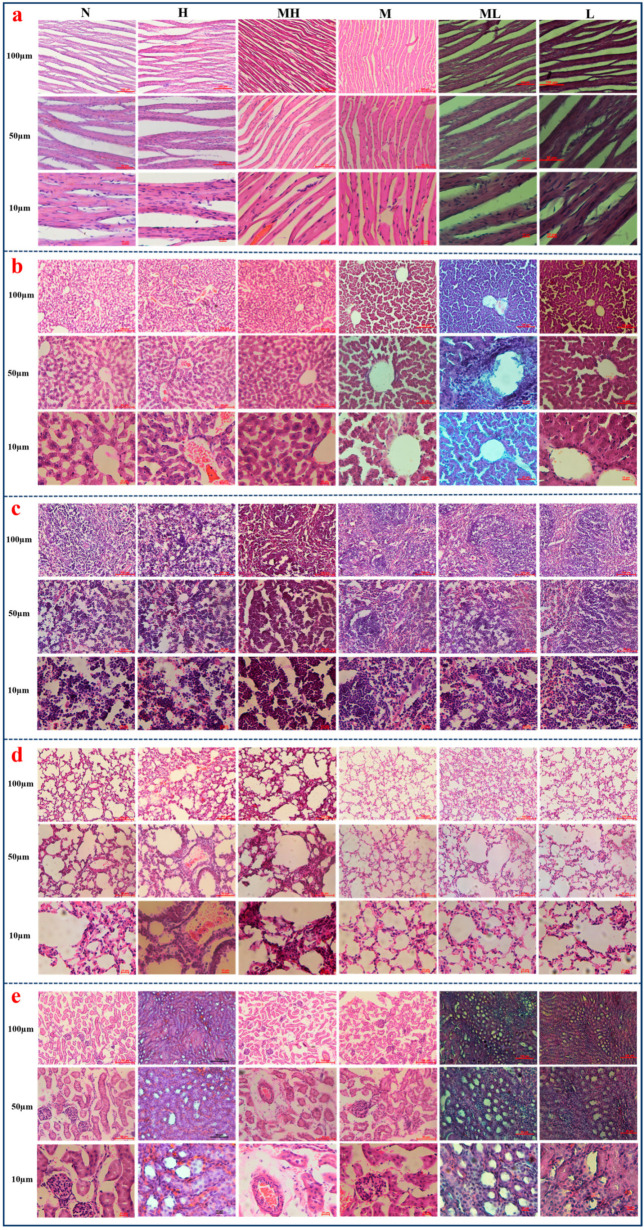
Typical HE (hematoxylin-eosin) staining results of heart, liver, spleen, lung, and kidney of mice in different groups. The experimental group was divided into six groups according to the different doses. N represents the control group; H represents the high group; MH represents the medium-high group; M represents the medium group; ML represents the medium-low group; L represents the low group. (**a**–**e**) represent different organs. (**a**) for heart, (**b**) for liver, (**c**) for spleen, (**d**) for lungs, and (**e**) for kidney.

The body weight of the surviving mice, measured on the 7th and 14th day, demonstrated a statistically insignificant upward trend (*p* > 0.05) (Supplementary Figure S3A). To evaluate the potential toxicity of MASM on the visceral organs of mice, we calculated and depicted the indices of heart, liver, spleen, lung, and kidney viscera (as the ratio of visceral weight to body weight) in Supplementary Figure S3B. Notably, no significant alterations in the viscera indices were observed when comparing the control group with groups treated with various dosages of MASM. Furthermore, all organs examined in both the MASM-treated groups and the control group exhibited well-preserved structures without any signs of inflammatory cell infiltration. The myocardial fibers and myocytes displayed a regular and organized arrangement. However, as depicted in Figure 7a, a noticeable increase in myocardial fiber density was observed in the high and medium-high-dose MASM groups, whereas the remaining treatment groups exhibited consistency with the control group, indicating no statistically significant alterations. Concurrently, the high and medium-high-dose MASM groups exhibited a noteworthy escalation in the number of hepatocytes (Figure 7b), accompanied by portal area bleeding, while there were no significant changes in the other administration groups. Moreover, the groups administered with high and medium-high doses of MASM demonstrated a slight increase in the count of splenic sinusoids and lymph nodes in comparison to the control group (Figure 7c). Additionally, in the high-dose MASM group, as depicted in Figure 7d, the dimensions of the lung alveolar cavity were observed to be widened, accompanied by hemorrhaging, while the alveolar epithelial cells exhibited indications of proliferation. Conversely, from the medium-dose group to the low-dose group, the alveolar wall displayed a slight thinning, and the pathological alterations were minimal. The results depicted in Figure 7e demonstrate that the kidney of the high-dose MASM group exhibited proliferation and dense arrangement, accompanied by evident bleeding. Similarly, the renal interstitial blood vessels in the medium-high and medium-dose MASM groups displayed proliferation and dilation. These findings indicated that there were no substantial pathological alterations in any of the administration groups, particularly in the medium, medium-low, and low-dose groups. Furthermore, the surviving mice exhibited a healthy condition and did not exhibit any significant behavioral changes throughout the 14-day duration.

#### 2.4.3. Effects of MASM on the Liver and Kidney Functions of Mice

The biochemical parameters of the liver and kidney in mice were assessed to further explore the potential toxicity of MASM. Examination of the data, as illustrated in Supplementary Figure S3C,D, revealed no statistically significant differences in the activities of aspartate aminotransferase (AST), aspartate transaminase (ALT), and alkaline phosphate (ALP), as well as the levels of total protein (TP) and albumin (ALB) in the plasma of mice treated with MASM compared to the control group (*p* > 0.05). Similarly, the levels of urea (UREA) and creatinine (CREA) in the plasma of mice treated with MASM were found to be statistically indistinguishable from those of the control group (*p* > 0.05).

## 3. Discussion

Currently, there remains the absence of an adequate treatment for liver cancer, and it has been postulated that impeding the transition from hepatitis to cancer is a vital strategy in averting the onset of liver cancer. MASM, a novel compound derived from matrine, has exhibited notable anti-inflammatory properties and negligible cytotoxicity. Prior investigations have indicated that MASM can effectively counteract liver fibrosis through diverse mechanisms [8]. However, there exists a scarcity of data pertaining to the pharmacokinetics, toxicity, and metabolites of MASM. It is widely acknowledged that the experimental evaluation of pharmacokinetic properties and toxicity is indispensable in the process of drug discovery. A substantial portion of tested compounds (approximately 40%) are rejected during phase I clinical trials due to insufficient preclinical pharmacokinetic properties [9]. Consequently, the current study aimed to estimate the pharmacokinetic properties and acute toxicity of MASM. Firstly, the plasma drug concentration–time curves of MASM in rats were investigated through three distinct administration modes, and the pharmacokinetic parameters were determined using a non-compartmental model, which is particularly suitable for new chemical entities that lack prior pharmacokinetic data [10]. It was observed that the half-life of MASM was consistent at approximately 10 h across all three routes of administration, suggesting a slow elimination rate of MASM in vivo. Notably, the peak plasma drug concentration was achieved at approximately 1.3 h following intragastric administration, indicating that MASM could be absorbed quickly after oral administration. According to previous research conducted by Zhang et al. [11], the plasma concentration of matrine reached its peak at 2.1 h after oral administration. The *T_max_* of MASM was shorter compared to matrine, allowing it to produce pharmacological effects more rapidly. Additionally, the plasma concentration–time curves for all administration groups demonstrated a shoulder peak, especially via intragastrical administration. This phenomenon is commonly observed in drugs that undergo enterohepatic circulation. Concerning the tissue distribution outcomes obtained from our experimental investigations, it was observed that following the initial hour, the concentration of MASM in the liver, kidney, and intestine tissues surpassed that of other organs. Subsequently, at the 2 h and 24 h marks, a relatively elevated concentration of MASM was detected in the spleen and intestine. These findings potentially indicate the existence of enterohepatic circulation for MASM, suggesting the possibility of reabsorption of a portion of the compound in the intestine. Consequently, the absolute bioavailability of MASM was determined to be 44.50% in the present study, which represents a significant increase compared to the bioavailability of matrine (18.5%) as reported by Yang et al. [12]. The apparent distribution volume of MASM was 84.74, 69.43, and 36.61 L/kg for intraperitoneal, intragastric, and intravenous administration, respectively. These values indicate a widespread distribution of MASM in different tissues and organs, a finding that is further supported by the results of tissue distribution experiments.

Secondly, the concentrations of MASM in multiple organs (heart, liver, spleen, lung, kidney, brain, muscle, intestine, and stomach) were assessed at three different time intervals within 24 h. The presence of MASM was detected in various tissues at 1, 2, and 24 h after administration, indicating a widespread distribution of MASM in rat tissues following its entry into the body. Specifically, the liver, kidney, and spleen exhibited higher accumulations of MASM compared to other tissues, potentially due to the metabolic and excretory processes associated with MASM or the significant blood perfusion in these organs.

Thirdly, the in vivo metabolites of MASM were also estimated in our present study. To enhance our comprehension of these metabolites, we initially postulated their potential structures by considering the established metabolic pathways and the chemical composition of MASM. Previous research [13] has suggested that alkaloids often undergo specific cleavage patterns, leading to the generation of fragment ions such as [M+H]^+^, [M+H-H_2_O]^+^, [C_10_H_16_N]^+^, and [C_10_H_14_N]^+^. These fragment ions may be associated with the presence of nitrogen-containing heterocycles within the molecular structure [14]. In our study, we observed that MASM in rats primarily underwent phase Ⅰ metabolic reactions, specifically oxidation, reduction, and hydroxylation. Through the utilization of primary and secondary mass spectrometry data, standard reference, and deuterium-labeled MASM, we were able to identify a total of 10 metabolites. These metabolites include matrine, sophorine, oxysophocarpine, and oxymatrine, which are natural compounds known to be present in *S. alopecuroide*. It is important to note that previous studies have reported varying degrees of hepatotoxicity associated with the aforementioned metabolites [15,16]. Therefore, given the in vivo metabolites of MASM observed in our current study and their corresponding toxicity, it is advisable to conduct additional structural optimization to mitigate the toxicity of MASM.

Finally, an acute toxicity test was performed to further evaluate the safety of MASM. Generally, a significant decline in body weight and a decrease in the weight of internal organs are regarded as pivotal indicators of animal health deterioration and direct measures of toxicity [17]. Our in vivo findings demonstrated that the body weight of all experimental mice exhibited a consistent increase over 14 days, while the organ-to-body weight ratio remained unchanged. Furthermore, the analysis of biochemical markers, such as AST, ALT, ALP, TP, ALB, UREA, and CREA, revealed no statistically significant differences between the control group and the various dosages of MASM groups. These collective findings strongly suggest that the administration of MASM had minimal or no adverse effects on the body weight, organ weight, and biochemical markers of the mice, thus indicating its non-toxic or low-toxic nature. During the 14-day observation period, no discernible alterations in fur color and skin condition were observed among the surviving mice. HE staining did not reveal any significant pathological changes in mice from each administration group, particularly in the low-dose (31.1 mg/kg), medium-low-dose (51.84 mg/kg), and medium-dose (86.4 mg/kg) MASM groups. Our experimental findings determined the LD_50_ of MASM in mice to be 94.25 mg/kg, which was slightly higher than the reported LD_50_ of matrine in the literature [18], suggesting that the toxicity of MASM is comparatively lower than that of matrine.

## 4. Materials and Methods

### 4.1. Chemicals and Reagents

MASM and deuterium-labeled MASM (DMASM) were prepared by the Second Military Medical University with a purity of higher than 98% (the results of NMR and MS are presented in Supplementary File S1); matrine (CAS 519-02-8, AF7033117), oxymatrine (CAS 16837-52-8, AF7112226), sophocarpine (CAS 6483-15-4, AF7122506), oxysophocarpine (CAS 26904-64-3, AF7112910), and aloperine (CAS 56293-29-9, AF8062714) were purchased from Chengdu AIFA Biotechnology Co., Ltd. (Chengdu, China) with purities all higher than 95%. Hematoxylin and eosin (Beijing Leagene Biotech. Co., Ltd., Beijing, China). LC grade methanol (Thermo Fisher Technology Co., Ltd., Shanghai, China), acetonitrile (Merck KGaA, Darmstadt, Germany), and formic acid (MREDA, Portland, ME, USA). 4% Paraformaldehyde (Beijing Leagene Biotech. Co., Ltd., Beijing, China). Phosphate buffered saline (Beijing Leagene Biotech. Co., Ltd., Beijing, China). All the other reagents were of analytical grade.

### 4.2. UHPLC-QQQ-MS/MS Conditions

UHPLC analysis was performed using a Thermo Accela AS system, employing an ACQUITY UPLC^®^BEH C18 (Waters Corporation, Wexfords, Ireland) (1.7 µm, 2.1 × 100 mm) column and a binary solvent system consisting of water containing 0.1% formic acid as mobile phase A and acetonitrile as mobile phase B. For pharmacokinetic evaluation, the gradient elution was set as follows: 97% A from 0 to 2 min, 97–70% A from 2 to 3 min, 70–20% A from 3 to 3.5 min, 20% A from 3.5 to 3.6 min, 20–97% A from 3.6 to 3.7 min, and 97% A from 3.7 to 5 min. For the metabolic test, the gradient elution in full scan mode was set as follows: 97% A from 0 to 2 min, 97–70% A from 2 to 4 min, 70–20% A from 4 to 6 min, 20% A from 6 to 7 min, 20–97% A from 7 to 8 min, and 97% A from 8 to 10 min. The flow rate was 0.2 mL/min, with a temperature of 40 °C maintained and an injection volume of 5 μL. The QQQ-MS/MS analysis was conducted using a Thermo TSQ QUANTUM ACCESS MAX system equipped with an electrospray ionization (ESI) source. The spray voltages were set at 4.0 kV and 2.5 kV for positive and negative ion modes, respectively; capillary and vaporizer temperatures were 320 °C and 350 °C, respectively; sheath and aux gas pressures were 35 and 10 psi, respectively.

### 4.3. Sample and Standard Solution Preparation

#### 4.3.1. Sample Solution Preparation

A precisely measured MASM sample weighing 400 mg was dissolved in normal saline to create aqueous solutions with concentrations of 8.0, 6.0, and 1.5 mg/mL for intraperitoneal, oral, and intravenous administrations at doses of 80 mg/kg, 60 mg/kg, and 15 mg/kg, respectively.

#### 4.3.2. Standard Solution and Internal Standard Solution Preparation

Standard stock solutions of MASM and aloperin (used as an internal standard) were prepared in methanol at a concentration of 1.0 mg/mL, respectively. The MASM stock solution was then diluted with methanol to generate a series of working standard solutions with concentrations ranging from 0.2 to 40.0 μg/mL. The IS stock solution was diluted with acetonitrile to a concentration of 10 ng/mL. Subsequently, 10 μL of each working standard solution, along with 300 μL of IS, were added to 90 μL of plasma or tissue homogenate derived from various biological matrices, including the heart, liver, spleen, lung, kidney, stomach, muscle or brain. Then, the resulting mixture underwent centrifugation at 13,000 rpm for 10 min, yielding a supernatant that was utilized for subsequent analysis. All the solutions were kept at 4 °C before use.

### 4.4. Animal Studies

The animal experiment strictly adhered to the laboratory animal principles and was conducted in accordance with the guidelines set forth by the Bioethics Committee of Ningxia Medical University (license number: IACUC-NYLAC-2022-142). A total of 60 three-month-old ICR mice for toxicity investigation (30 males and 30 females, weighing 22 ± 4 g) and 110 three-month-old Sprague-Dawley rats (55 males and 55 females, weighing 220 ± 20 g) for the pharmacokinetic experiment were provided by the Experimental Animal Center of Ningxia Medical University (License number: SCXK (Ning)-2020-0001). All animals were acclimated for 1 week in a clean environment with constant temperature and humidity (12 h/12 h light/dark cycle at 25 ± 2 °C and relative humidity of 65 ± 5%) and were fed and given water ad libitum.

#### 4.4.1. Blood Concentration–Time Profiles and Metabolite Experiments of MASM

Seventy Sprague-Dawley rats were fasted overnight before dosing, with free access to water. Subsequently, the rats were randomly and equally divided into 7 groups: the control groups received 3 different routes of administration: intravenous, intraperitoneal, and intragastric (NS, saline 1 mL/100 g); MASM intraperitoneal group (A, i.p. 80 mg/kg), MASM intragastrical group (B, i.g. 60 mg/kg), MASM intravenous group (C, i.v. 15 mg/kg), and DMASM intragastrical group (D, i.g. 60 mg/kg). It is important to note that the recommended clinical dose of matrine injection is 0.15 g, which is equivalent to a dosage of 15 mg/kg in rats. MASM was administered via tail vein injection at a dosage of 1 mL/100 g for intravenous dosing, while for intraperitoneal dosing, MASM was injected at the lower-right side of the abdomen of rats. Blood samples (approximately 0.3 mL) were collected from the eye socket at specific time intervals (0.083, 0.167, 0.25, 0.5, 1, 1.5, 2, 4, 6, 9, 12, and 24 h) after administration for each group. These samples were then placed in centrifuge tubes containing heparin sodium, subjected to centrifugation at 4000 rpm for 15 min, and the resulting supernatant was collected and stored at −80 °C until further analysis. During the analysis of the plasma sample, protein precipitation was carried out using a plasma-to-acetonitrile ratio of 1:3, and 5μL of the resulting supernatant was injected. The developed UHPLC-QQQ-MS/MS analysis method underwent a series of validations, and the peak area of the plasma sample was obtained employing the Xcalibur 2.2 data-processing system. The concentrations of MASM in plasma were then determined using linear regression equations. Subsequently, the in vivo pharmacokinetic parameters were calculated using the non-compartment model in DAS 3.0 software. Blood concentration–time curves were generated using Prism 8.0 (GraphPad Software Inc., San Diego, CA, USA).

#### 4.4.2. Tissue Distribution of MASM

A total of 36 Sprague-Dawley rats (18 male and 18 female) were randomly and equally assigned to 6 groups, with 3 male and 3 female rats in each group. Among these groups, 3 groups were administrated MASM intragastrically at a dosage of 60 mg/kg, while the remaining 3 groups received intraperitoneal administration of MASM at a dosage of 80 mg/kg. The rats in each group were euthanized at three different time points: 1 h, 2 h, and 24 h after administration. Subsequently, various tissues, including the heart, liver, spleen, lung, kidney, stomach, muscle, and brain, were promptly collected and weighed. These tissues were then homogenized using acetonitrile at a ratio of 1:10 and stored at a temperature of −80 °C until further analysis. During the analysis of the tissue sample, the protein was precipitated following the tissues: acetonitrile with a ratio of 1:3 and a volume of 5μL of the resulting supernatant were injected. The UHPLC-QQQ-MS/MS analysis method that was developed, underwent a comprehensive validation process, and the concentration of MASM in each tissue was determined using linear regression equations. Bar graphs were generated by applying Prism 8.0 (GraphPad Software Inc., San Diego, CA, USA).

#### 4.4.3. Acute Toxicity of MASM in Mice

A total of 60 ICR mice were equally and randomly divided into 6 groups, namely the control group (N, saline 1 mL/100 g), MASM high group (H, 240 mg/kg), MASM medium-high group (MH, 144 mg/kg), MASM medium group (M, 86.4 mg/kg), MASM medium-low group (ML, 51.84 mg/kg), and MASM low group (L, 31.10 mg/kg). All mice were administered MASM intravenously. The body weight of each mouse was measured on days 7 and 14. On the 14th day, all mice were euthanized, and the heart, liver, spleen, lung, and kidney were collected and weighed immediately. Serum was collected from the eyeballs for further biochemical analysis, and a small portion of each tissue and organ was fixed in 4% Faure Marin solution for histopathological examination. Bar graphs were generated applying Prism 8.0 (GraphPad Software Inc., San Diego, CA, USA), and HE staining samples were captured using the Leica image system.

### 4.5. Method Validation

The developed LC-MRM-MS/MS analysis method was validated to ensure its reliability and stability. The validation process encompassed specificity, linearity, accuracy, precision, extraction recovery, matrix effects, and stability. The detailed procedures can be found in Supplementary File S2.

### 4.6. Statistical Analysis

The results were described as mean ± SD (Standard Deviation) and were analyzed using SPSS software (version 18.0, IBM SPSS Statistics, IBM Corp., Armonk, NY, USA).

## 5. Conclusions

In summary, this study presents the first comprehensive evaluation of the pharmacokinetic characteristics, tissue distribution, metabolites, and acute toxicity of MASM. The findings demonstrated that MASM exhibited significantly higher bioavailability compared to the lead compound, matrine. Moreover, the substantial concentration of MASM in liver tissue suggests its potential efficacy in the treatment of liver disease. Notably, the observed lower acute toxicity of MASM in comparison to that of matrine indicates the superiority of MASM as a structural modifier of matrine. Furthermore, our data provide a basis for further understanding the structure–activity relationships between matrine and MASM.

## Figures and Tables

**Table 1:** Main non-compartmental pharmacokinetic parameters of MASM after intravenous (15 mg/kg), intraperitoneal (80 mg/kg), or intragastrical (60 mg/kg) administration (*n* = 6, mean ± SD (Standard Deviation)).

Parameter	Unit	i.p.	i.g.	i.v.
AUC_(0–t)_	mg/L·h	12.43 ± 5.32	10.86 ± 4.78	4.28 ± 2.40
AUC_(0–24)_	mg/L·h	15.58 ± 5.61	13.70 ± 7.55	5.41 ± 3.22
*t*_1/2_	h	9.97 ± 3.95	10.25 ± 4.95	10.51 ± 8.84
*T_ma_*_x_	h	0.21 ± 0.04	1.31 ± 0.53	0.17 ± 0.17
*V*_d_	L/Kg	84.74 ± 57.17	69.43 ± 22.58	36.61 ± 17.83
*CL*	L/h/Kg	5.73 ± 2.03	5.60 ± 2.94	4.30 ± 3.47
*C* *_max_*	mg/L	2.21 ± 0.93	1.38 ± 0.57	0.78 ± 0.54
F(%)		54.01 ± 19.44	44.50 ± 12.26	

**Table 2:** Metabolites profiling of MASM.

Metabolites	Method of Administration	Molecular Formula	*t*_R_/mins	*m*/*z*		Fragment Ions	Structure
				Theoretical Value	Actual Value		
M1 (demethoxy MASM)	i.p. i.g.	C_15_H_24_N_2_S	1.57	265.17	265.06	230.83, 150.0, 136.17, 96.11, 122.28, 148.04	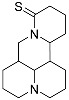
M2	i.p. i.g.	C_15_H_22_N_2_S	5.59	263.15	262.98	228.84, 149.86, 136.08, 122.41, 134.01, 179.29	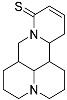
M3 (sophocarpine)	i.p. i.g.	C_15_H_22_N_2_O	1.58	247.17	247.14	179.18, 150.36, 161.93, 136.16, 147.69	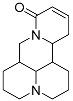
M4 (oxysophocarpine)	i.p. i.g.	C_15_H_22_N_2_O_2_	1.58	263.17	263.06	245.67, 150.15, 148.36, 136.35,	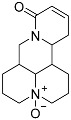
M5 (oxymatrine)	i.p. i.g.	C_15_H_24_N_2_O_2_	1.57	265.18	265.27	150.00, 148.04, 136.17,	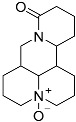
M6	i.p. i.g.	C_16_H_29_N_3_S	1.61	296.21	295.80	263.16, 265.42, 239.07, 110.04	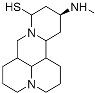
M7	i.p. i.g.	C_16_H_25_N_3_S	1.60	280.18	280.16	203.5, 179.37, 149.85, 162.03 147.75	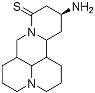
M8	i.g.	C_16_H_25_N_3_S	1.57	292.18	292.12	111.92, 135.85, 150.16, 178.77	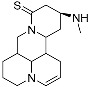
M9	i.g.	C_16_H_27_N_3_OS	1.57	310.19	309.95	110.02, 176.66, 187.99, 262.69	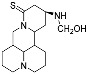
M10	i.g.	C_16_H_25_N_3_OS	1.62	308.17	308.36	122.10, 148.16, 192.93, 233.05,	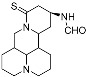

## Data Availability

The original contributions presented in the study are included in the article/Supplementary Materials; Further inquiries can be directed to the corresponding author.

## Supplementary Materials

The following supporting information can be downloaded at: https://www.mdpi.com/article/10.3390/molecules29020297/s1, Figure S1: Representative EIC of different plasma samples; Figure S2: Representative EIC of different liver samples. Figure S3: Results of body weight and viscera index as well as the levels of biochemical parameters after intravenous administration of MASM; Supplementary File S1: 1H NMR and MS of MASM and DMASM; Supplementary File S2: Pharmacokinetic experiment; Supplementary File S3: Calibration curves of MASM in each control tissue; Supplementary File S4: The identification process of metabolites of MASM.

## Abbreviations

MT	matrine
ALB	albumin
ALP	alkaline phosphatase
ALT	aspartate transaminase
AST	aspartate aminotransferase
AUC	area under the curve
CL	clearance
*C* *_max_*	peak concentration
CREA	creatinine
EIC	Extracted ion chromatogram
F	absolute intragastrical bioavailability
HE	hematoxylin-eosin
IS	internal standard
GFAP	Glial fibrillary acidic protein
LD_50_	medium lethal dose
SD	Standard Deviation
*T_max_*	peak time
*t*_1/2_	half-life time
TP	total protein
UREA	urea
V	apparent volume of distribution

## References

[1] Wang M.C. (2020). The Mechanism of the Progress from Hepatitis to Hepatocellular Carcinoma through the Excretion of Exosomes Containing DANCR by the Liver Cells. Ph.D. Thesis.

[2] Cao W., Chen H.D., Yu Y.W., Li N., Chen W.Q. (2021). Changing profiles of cancer burden worldwide and in China: A secondary analysis of the global cancer statistics 2020. Chin. Med. J..

[3] Hewitt D.B., Rahnemai-Azar A.A., Pawlik T.M. (2021). Potential experimental immune checkpoint inhibitors for the treatment of cancer of the liver. Expert Opin. Inv. Drug..

[4] Wang X., Lin H., Zhang R. (2017). The clinical efficacy and adverse effects of interferon combined with matrine in chronic hepatitis b: A systematic review and Meta-Analysis. Phytother. Res..

[5] Li X., Tang Z.W., Wen L., Jiang C., Feng Q.S. (2021). Matrine: A review of its pharmacology, pharmacokinetics, toxicity, clinical application and preparation researches. J. Ethnopharmacol..

[6] Feng Y., Ying H., Qu Y., Cai X., Xu M., Lu L. (2016). Novel matrine derivative MD-1 attenuates hepatic fibrosis by inhibiting EGFR activation of hepatic stellate cells. Protein Cell.

[7] Li J., Xu J., Lu Y., Qiu L., Xu W., Lu B., Hu Z., Chu Z., Chai Y., Zhang J. (2016). MASM, a Matrine Derivative, Offers Radioprotection by Modulating Lethal Total-Body Irradiation-Induced Multiple Signaling Pathways in Wistar Rats. Molecules.

[8] Zou Y., Sarem M., Xiang S., Hu H., Xu W., Shastri V.P. (2019). Autophagy inhibition enhances Matrine derivative MASM induced apoptosis in cancer cells via a mechanism involving reactive oxygen species-mediated PI3K/Akt/mTOR and Erk/p38 signaling. BMC Cancer.

[9] Gutiérrez-Sánchez M., Romero-Castro A., Correa-Basurto J., Rosales-Hernández M.C., Padilla-Martínez I.I., Mendieta-Wejebe J.E. (2021). Preclinical pharmacokinetics and acute toxicity in rats of 5-{[(2E)-3-Bromo-3-carboxyprop-2-enoyl] amino}-2-hydroxybenzoic acid: A novel 5-Aminosalicylic acid derivative with potent Anti-Inflammatory activity. Molecules.

[10] Sehgal P., Colombel J.F., Aboubakr A., Narula N. (2018). Systematic review: Safety of mesalazine in ulcerative colitis. Aliment. Pharm. Ther..

[11] Zhang L., Liu W., Zhang R., Wang Z., Shen Z., Chen X., Bi K. (2008). Pharmacokinetic study of matrine, oxymatrine and oxysophocarpine in rat plasma after oral administration of Sophora flavescens Ait. Extract by liquid chromatography tandem mass spectrometry. J. Pharmaceut. Biomed..

[12] Yang Z., Gao S., Yin T., Kulkarni K.H., Teng Y., You M., Hu M. (2010). Biopharmaceutical and pharmacokinetic characterization of matrine as determined by a sensitive and robust UPLC–MS/MS method. J. Pharmaceut. Biomed..

[13] Li X.N. (2019). Rapid Analysis of Migrating Components and Metabolites of Siwei TuMuxiang San in the Blood Based on HPLC-Q-Exactive-MS/MS. Master’s Thesis.

[14] Han F.M., Zhu M.M., Chen H.X., Chen Y. (2009). Identification of sophoridine and its metabolites in rat urine by liquid Chromatography-Tandem mass spectrometry. Anal. Lett..

[15] Shi H.J., Song H.B., Wang L., Xiao S.X., Bo K.P., Ma W. (2018). The synergy of diammonium glycyrrhizinate remarkably reduces the toxicity of oxymatrine in ICR mice. Biomed. Pharmacother..

[16] Lu Z.G., Li M.H., Wang J.S., Wei D.D., Liu Q.W., Kong L.Y. (2014). Developmental toxicity and neurotoxicity of two matrine-type alkaloids, matrine and sophocarpine, in zebrafish (Danio rerio) embryos/larvae. Reprod. Toxicol..

[17] Zhang Y.X., Zhang Y.M., Han Y.M., Tian Y., Wu P.C., Xin A.Y., Wei X.N., Shi Y.B., Zhang Z.C., Su G. (2020). Pharmacokinetics, tissue distribution, and safety evaluation of a ligustilide derivative (LIGc). J. Pharm. Biomed. Anal..

[18] Dai W., Qian L., Wang L., Yang S., Zhou G. (2012). Toxicity study of matrine and oxymatrine in mice. Anhui Med. Pharm. J..

