# Supplementary material for: Pharmacokinetic, Tissue Distribution, Metabolite, and Toxicity Evaluation of the Matrine Derivative, (6aS, 10S, 11aR, 11bR, 11cS)-10-Methylaminododecahydro-3a, 7a-Diaza-benzo (de) Anthracene-8-thione
Source: Molecules. 2024 Jan 6;29(2):297. doi: 10.3390/molecules29020297 (PMC10820135; doi:10.3390/molecules29020297)
Supplement: Supplementary file 1 [file molecules-29-00297-s001.zip › Supplementary Figures S1-S3.pdf]

Supplementary Figure S1-S3

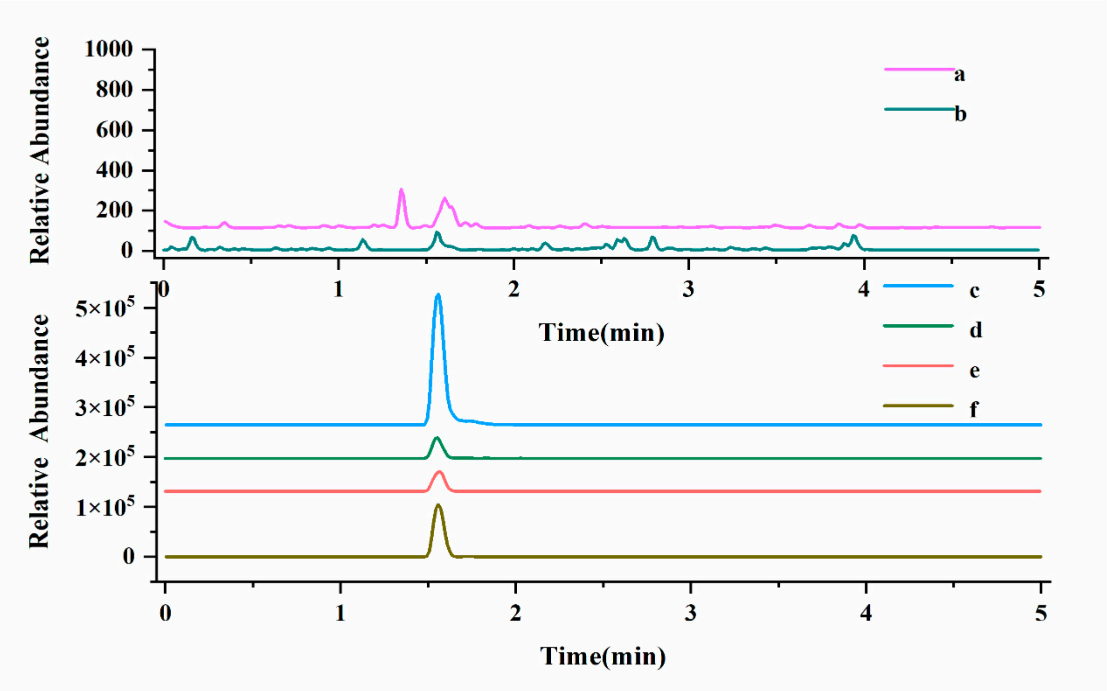

Supplementary Figure S1 Representative EIC of different plasma samples. a: EIC of MASM in control plasma; b: EIC of IS in control plasma; c: EIC of MASM in plasma of rats after administration; d: EIC of IS added in plasma of rats after administration of MASM; e: EIC of IS added in control plasma; f: EIC of MASM added in control plasma.

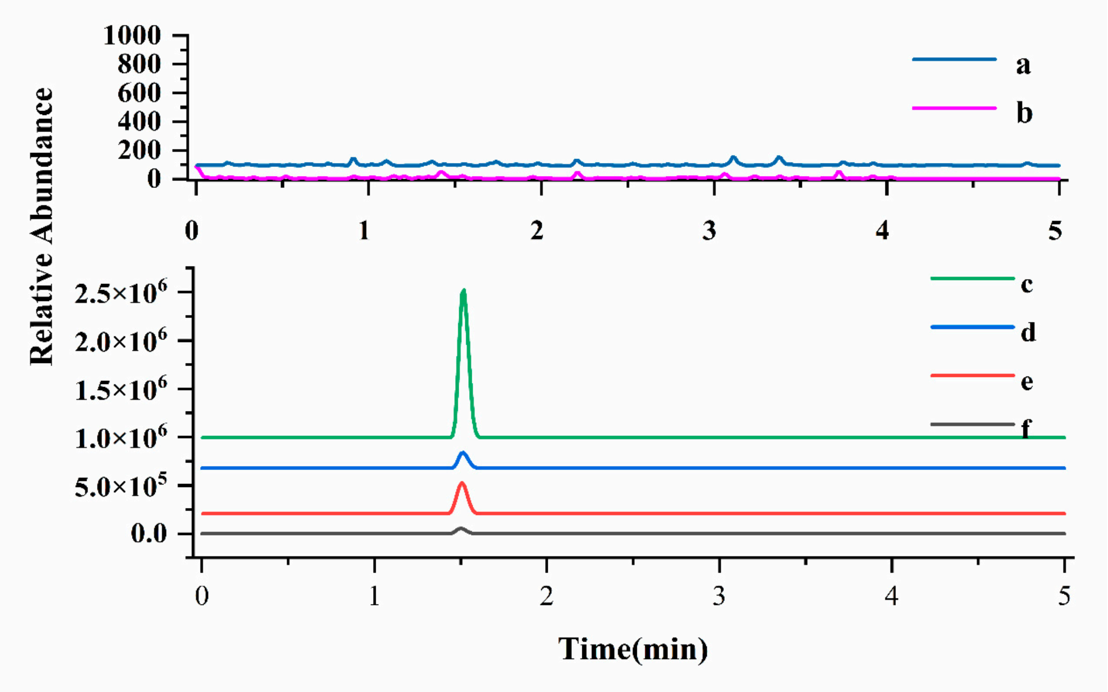

Supplementary Figure S2 Representative EIC of different liver samples. (a) a: EIC of MASM in control liver tissue; b: EIC of IS in control liver tissue; c: EIC of MASM in liver tissue of rats after administration; d: EIC of IS added in liver tissue of rats after administration of MASM; e: EIC of IS added in control liver tissue; f: EIC of MASM added in control liver tissue.

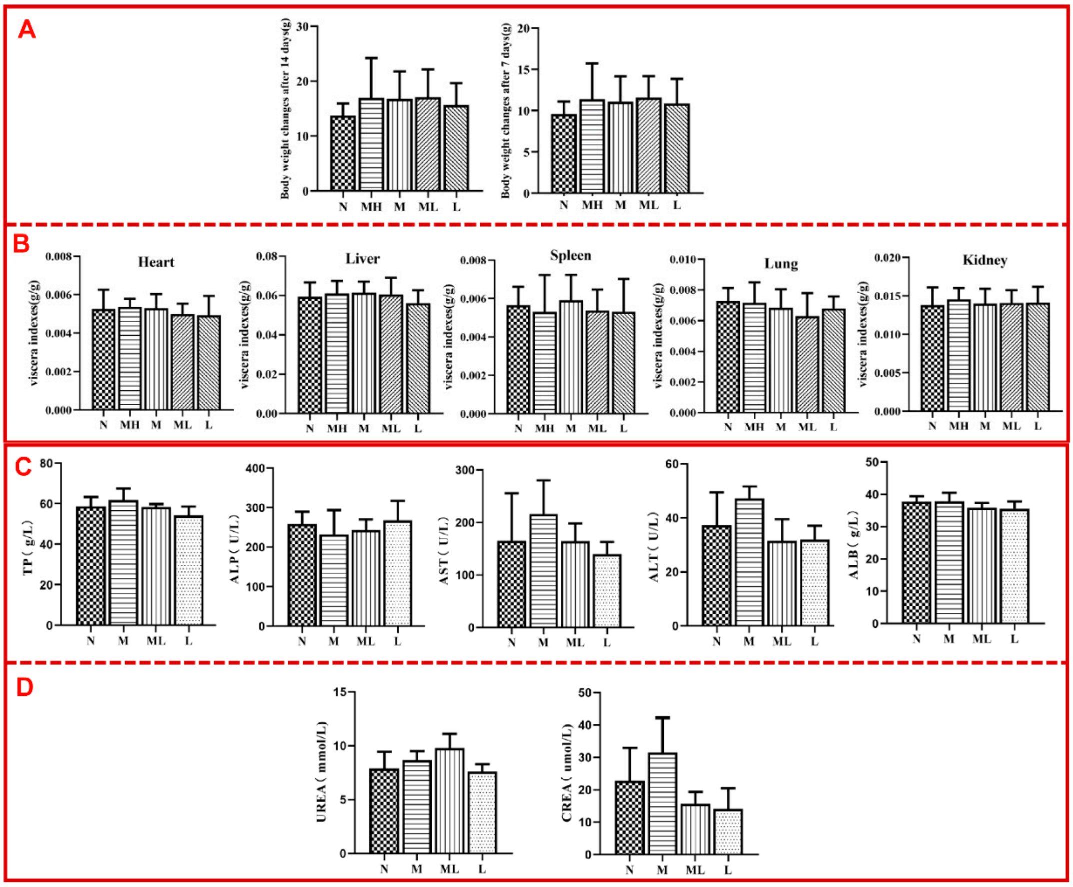

Supplementary Figure S3 Results of body weight and viscera index as well as the levels of biochemical parameters after intravenous administration of MASM. A: body weight changes of mice on the 7th and 14th day in different groups, respectively; B: the heart, liver, spleen, lung, and kidney viscera index of mice in different groups. C: levels of typical biochemical indicators for function of liver, including TP, ALP, AST, ALT, and ALB; D: levels of typical biochemical indicators for function of kidney, including UREA and CREA.
