# Supplementary material for: Pharmacokinetic, Tissue Distribution, Metabolite, and Toxicity Evaluation of the Matrine Derivative, (6aS, 10S, 11aR, 11bR, 11cS)-10-Methylaminododecahydro-3a, 7a-Diaza-benzo (de) Anthracene-8-thione
Source: Molecules. 2024 Jan 6;29(2):297. doi: 10.3390/molecules29020297 (PMC10820135; doi:10.3390/molecules29020297)
Supplement: Supplementary file 1 [file molecules-29-00297-s001.zip › Supplementary File S1.pdf]

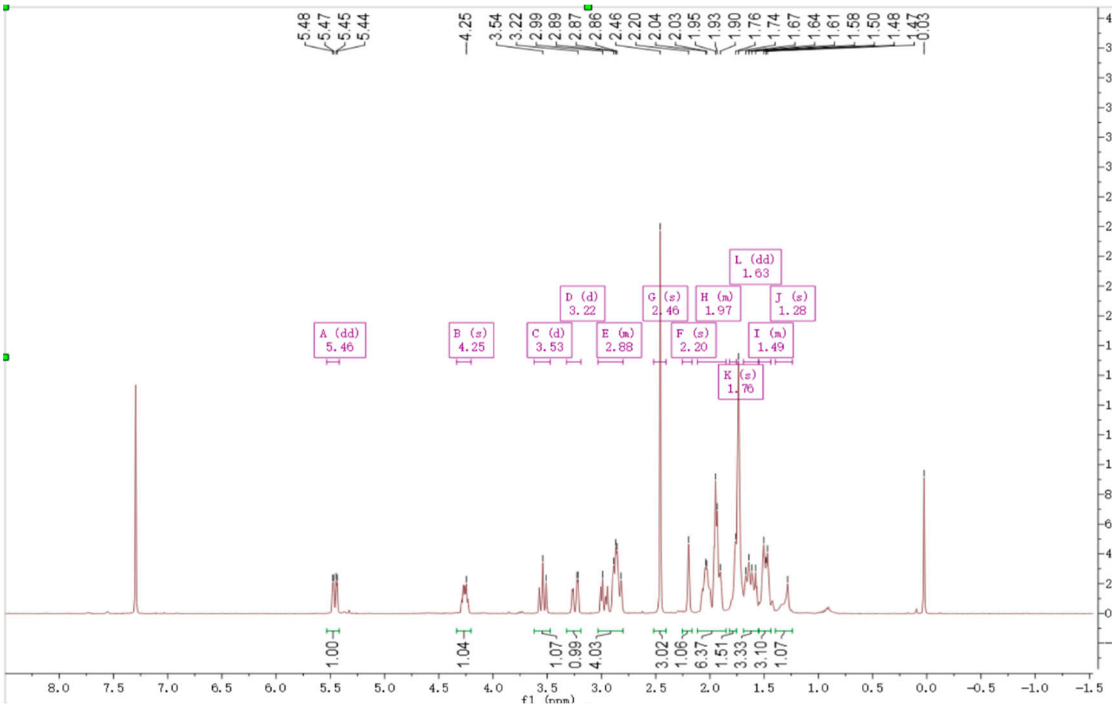

Display Report

Analysis Info

Analysis Name	D:\Data\2021MS\MZY\0521\M19_RB7_01_1942.d	Acquisition Date	5/21/2021 9:11:41 AM
Method	tune_low_20-400_pos-3min.m	Operator	gftang
Sample Name	M19	Instrument / Ser#	micrOTOF 10257
Comment			

Acquisition Parameter

Source Type	ESI	Ion Polarity	Positive	Set Nebulizer	1.0 Bar
Focus	Not active			Set Dry Heater	200 °C
Scan Begin	21 m/z	Set Capillary	2500 V	Set Dry Gas	4.0 l/min
Scan End	400 m/z	Set End Plate Offset	-500 V	Set Divert Valve	Waste

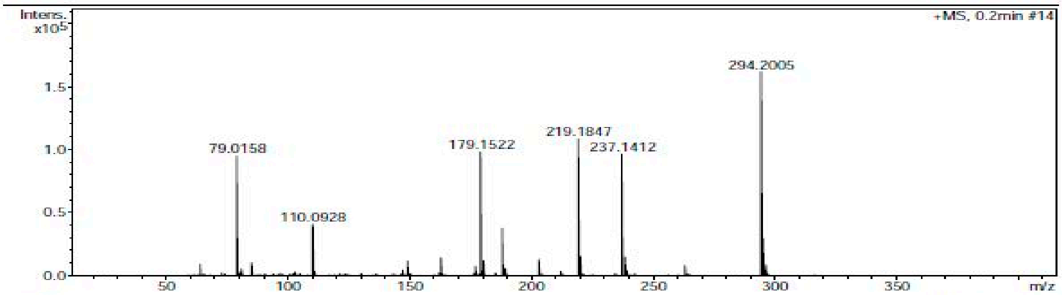

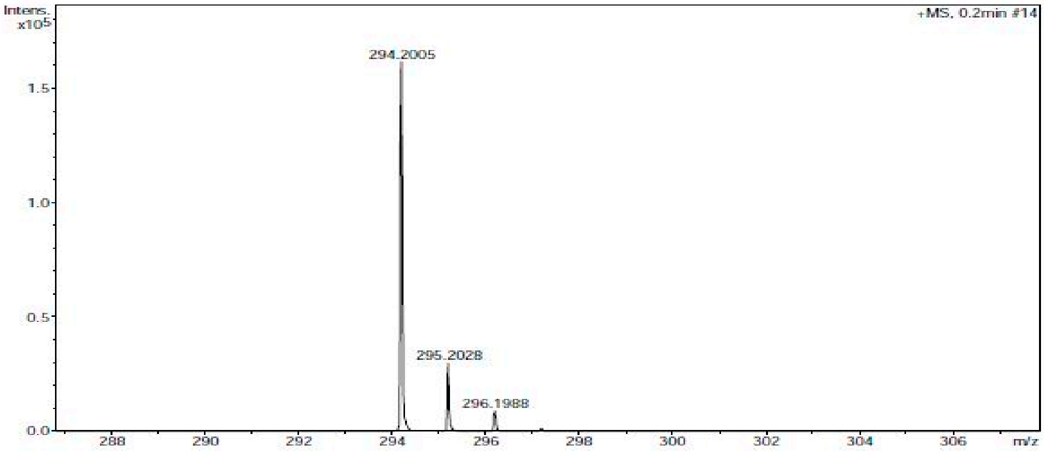

Bruker Compass DataAnalysis 4.0 printed: 5/21/2021 9:50:13 AM Page 1 of 1

Supplementary File S1 1H NMR and MS of MASM

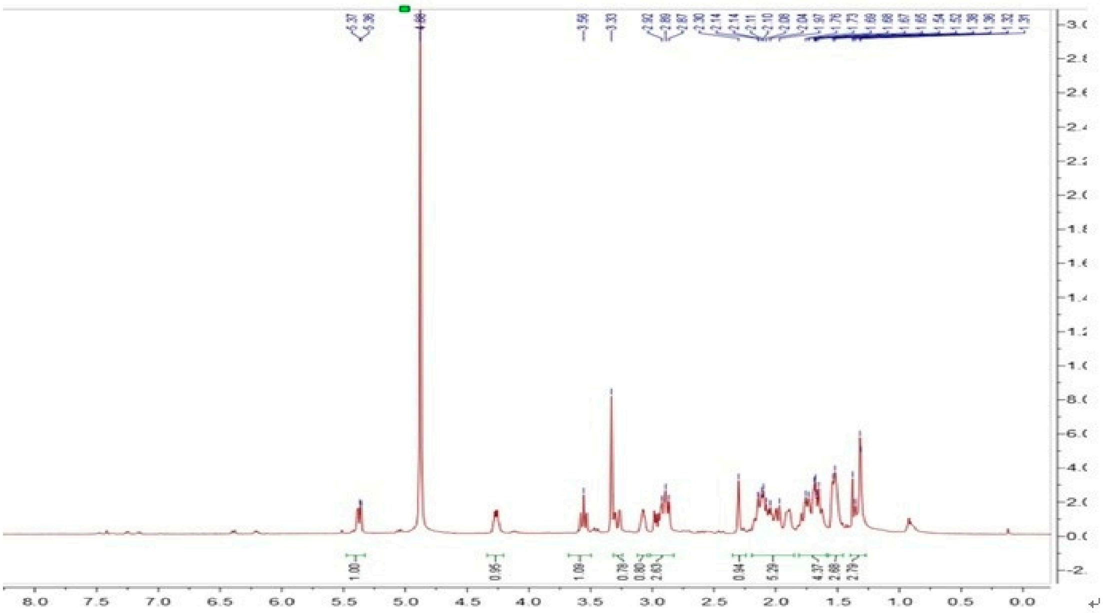

Generic Display Report

Analysis Info

Analysis Name	D:\Data\2021MS\MZY\1216\dm19-1_RB3_01_586.d	Acquisition Date	12/16/2021 9:59:11 AM
Method	tune_low_20-400_pos-6min.m	Operator	gftang
Sample Name	dm19-1	Instrument	micrOTOF
Comment			

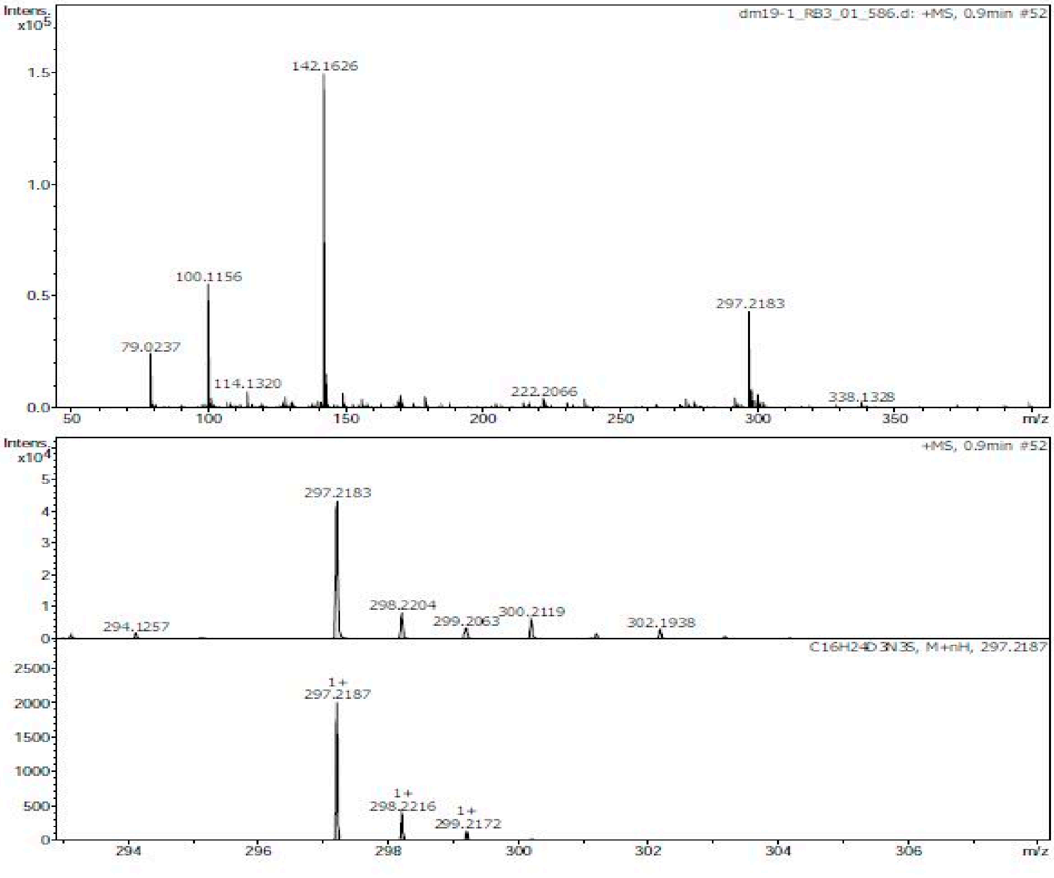

Bruker Compass DataAnalysis 4.1 printed: 12/16/2021 10:12:42 AM by: gftang Page 1 of 1

Supplementary File S1 1H NMR and MS of DMASM
