# Supplementary material for: Pharmacokinetic, Tissue Distribution, Metabolite, and Toxicity Evaluation of the Matrine Derivative, (6aS, 10S, 11aR, 11bR, 11cS)-10-Methylaminododecahydro-3a, 7a-Diaza-benzo (de) Anthracene-8-thione
Source: Molecules. 2024 Jan 6;29(2):297. doi: 10.3390/molecules29020297 (PMC10820135; doi:10.3390/molecules29020297)
Supplement: Supplementary file 1 [file molecules-29-00297-s001.zip › Supplementary File S2.pdf]

Supplementary File S2—Pharmacokinetic experiment

1. Methods

1.1 Preparation of internal and mixed standard solutions

A standard solution containing MASM at a concentration of 1.0 mg/ml was prepared by using methanol as the solvent. This standard solution was then diluted to obtain concentrations of 32 μg/ml, 20 μg/ml, and 4 μg/ml. To ensure quality control, 10 μl of each standard solution, along with 300 μl of the internal standard (IS), were mixed with 90 μl of plasma to create high, medium, and low concentration quality control (QC) samples, respectively.

1.2 Linearity

Six levels of standard solutions of MASM were added in blank plasma. The ratio of peak area of IS to that of the sample was denoted as Y, while the corresponding concentration was represented as X. The linear regression equation was established through regression calculation using the weight least-square linear regression method. The lower limit of quantification (LLOQ) was defined as the minimum point on the standard curve that could be accurately quantified with a precision and accuracy within ±20%.

1.3 Accuracy and precision

The accuracy and precision were expressed using the relative deviation (*RE*) and relative standard deviation (*RSD*), respectively, which HQC and MQC were required within ±15%, while LQC needed to be within ±20%. To assess the accuracy and precision of the method, six QC samples of MASM at three different concentrations were prepared and measured over three consecutive days, with subsequent calculation of the sample concentrations. The accuracy was expressed as relative deviation (*RE*), *RE*% = [(measured value - true value)/true value] × 100%. The intra-day precision was

determined by calculating the *RSD* of 6 samples analyzed on the same day, while the inter-day precision was determined by calculating the *RSD* of 3 analytical batches analyzed over 3 consecutive days.

1.4 Extraction recovery and matrix effect

The extraction recovery was in assessed by comparing the peak area of the blank plasma samples spiked with analytes and IS before and after extraction, as demonstrated in Eq. (1); and the matrix effect was evaluated by comparing the peak areas of processed blank plasma samples (with added analytes and IS solution) after extraction with the corresponding standard solutions, as presented in Eq. (2)

$$\text{Extraction recovery}=A_p/A_x\times100\% \qquad \text{Eq. (1)}$$

$$\text{Matrix effect}=A_x/A_s\times100\% \qquad \text{Eq. (2)}$$

A_p: 10 μl of high (H), medium (M), and low (L) concentration of standard solution and 300 μl of IS were added to 90 μl of blank plasma, respectively, then pre-processed by sample pretreatment method and injected to obtain the peak area A_p.

A_x: Supernatant was obtained by acetonitrile precipitation of protein in blank plasma. 10 μl of high, medium and low concentration standard solution and 300 μl of IS were added to 90 μl of the supernatant, respectively, and the peak area A_x was obtained by injection.

A_s: 10 μl of high, medium and low concentration of standard solution and 300 μl of IS solution were added into 90 μl of methanol solvent, then pre-processed by sample pretreatment method and injected to obtain the peak area A_s.

1.5 Stability

The stability of analytes in plasma was assessed by analyzing peak area in three different concentration QC samples. The long-term stability was evaluated by analyzing samples that had been stored at a temperature of -80°C for a duration of 30 days. The

freeze-thaw stability was assessed by analyzing samples that had undergone three freeze-thaw cycles, transitioning from a temperature of -80°C to 25°C. Post-preparative stability was assessed by analyzing samples that had been stored in the autosampler at 4°C for a duration of 8 h.

2. Results

The calibration curve was constructed by employing a linear least-squares regression model with $1/x^2$ as as the weighting factor, wherein the peak area ratios of the analyte to IS were plotted against concentrations. The resulting calibration curve exhibited a typical linear regression equation of y=0.05x–0.04 (*r*=0.9980). The MASM demonstrated excellent linearity, as evidenced by a correlation coefficient (*r*) exceeding 0.990 across the relevant concentration ranges (**Supplementary Table S1**). The lower limit of the quantitation (LLOQ) was determined to be 5.03 ng/ml.

Accuracy and precision were assessed at three levels of QC, and verified by *RSD* and *RE* (**Supplementary Table S2**), respectively. The maximum *RSD* of intra- and inter-day precision were 12.8 % and 16.2 %, respectively; while the accuracy was within ±15 % for all analytes. The results were met the acceptable criteria with satisfactory precision and accuracy.

The results of the extraction recovery and matrix effect of samples at three concentration levels were presented in **Supplementary Table S3**. The mean extraction recoveries of all analytes were ranged from 96.5% to 99.4%, while the mean matrix effects ranged from 95.7% to 97.2%. These findings indicated that the sample pretreatment method was suitable for achieving stable and high extraction recovery, and no significant endogenous interference was observed in the plasma samples.

The result of stability was summarized in **Supplementary Table S4**. No obvious degradation was found in the post-pretreatment, long-term and three freeze-thaw cycles

experiments, indicating that the analytes were stable in the plasma. The *RSD* was no more than 13.8%.

Table S1 Regression equations, linear ranges and correlation coefficients (*r*) of MASM

Analyte	Regression equations	Linear range (ng/mL)	*r*
MASM	y=0.05x-0.04	5.03~1006.25	0.9980

Table S2 Results of, accuracy and precision of plasma sample

QC samples	Accuracy		Precision (*RSD*%)	
	$\bar{x}$ ± SD	*RSD (*%)	Intra-	Inter-
HQC	95.7±5.4	5.6	5.6	9.9
MQC	100.5±4.4	4.3	4.4	12.5
LQC	101.5±12.0	11.8	12.8	16.2

Table S3 Results of matrix effect and extraction recovery of plasma sample

QC samples	extraction recovery		matrix effect	
	$\bar{x}$±SD	*RSD* (%)	$\bar{x}$±SD	*RSD* (%)
HQC	98.1±3.8	3.9	97.2±5.5	5.7
MQC	99.4±6.4	6.5	96.1±4.0	4.1
LQC	96.5±5.7	5.9	95.7±5.6	5.9

Table S4 Results of matrix effect and extraction recovery of plasma sample

QC samples	post-pretreatment *RSD* (%)	long-term *RSD* (%)	freeze-thaw cycle *RSD* (%)
HQC	6.0	7.1	7.7

MQC	6.9	12.5	9.8
LQC	11.7	12.5	13.8
