# Supplementary material for: Pharmacokinetic, Tissue Distribution, Metabolite, and Toxicity Evaluation of the Matrine Derivative, (6aS, 10S, 11aR, 11bR, 11cS)-10-Methylaminododecahydro-3a, 7a-Diaza-benzo (de) Anthracene-8-thione
Source: Molecules. 2024 Jan 6;29(2):297. doi: 10.3390/molecules29020297 (PMC10820135; doi:10.3390/molecules29020297)
Supplement: Supplementary file 1 [file molecules-29-00297-s001.zip › Supplementary File S3.pdf]

Supplementary File S3 Calibration curves of MASM in each control tissue

Tissues	Calibration curve	Correlation coefficient (*r*)
heart	y=0.29x＋1.32	0.9930
liver	y=0.15x+2.24	0.9935
spleen	y=0.38x+0.80	0.9823
lung	y=0.31x+1.21	0.9930
kidney	y=0.23x+0.68	0.9980
stomach	y=0.17x-0.27	0.9975
intestines	y=0.12x+0.93	0.9910
muscle	y=0.18x+0.73	0.9980
brain	y=0.09x-0.88	0.9935
