# Supplementary material for: Pharmacokinetic, Tissue Distribution, Metabolite, and Toxicity Evaluation of the Matrine Derivative, (6aS, 10S, 11aR, 11bR, 11cS)-10-Methylaminododecahydro-3a, 7a-Diaza-benzo (de) Anthracene-8-thione
Source: Molecules. 2024 Jan 6;29(2):297. doi: 10.3390/molecules29020297 (PMC10820135; doi:10.3390/molecules29020297)
Supplement: Supplementary file 1 [file molecules-29-00297-s001.zip › Supplementary File S4.pdf]

Supplementary File S4—The identification process of metabolites of MASM

The molecular formula of metabolite M3 was determined to be $C_{15}H_{22}N_2O$ (m/z 247.17 $[M+H]^+$), which exhibited a mass 16Da lower than that of M2 (m/z 263.15 $[M+H]^+$). It is speculated that oxygen atoms may substitute sulfur atoms in M2 structure. Notably, the major MS/MS fragment ions of M3 were observed at m/z 179.18, 150.36, 136.16, 147.69, and 161.93, which closely resemble the fragment ions of sophocarpine. The EIC diagram, MS/MS spectrum and EIC of standard reference were all showed in **Supplementary Figure F1.**

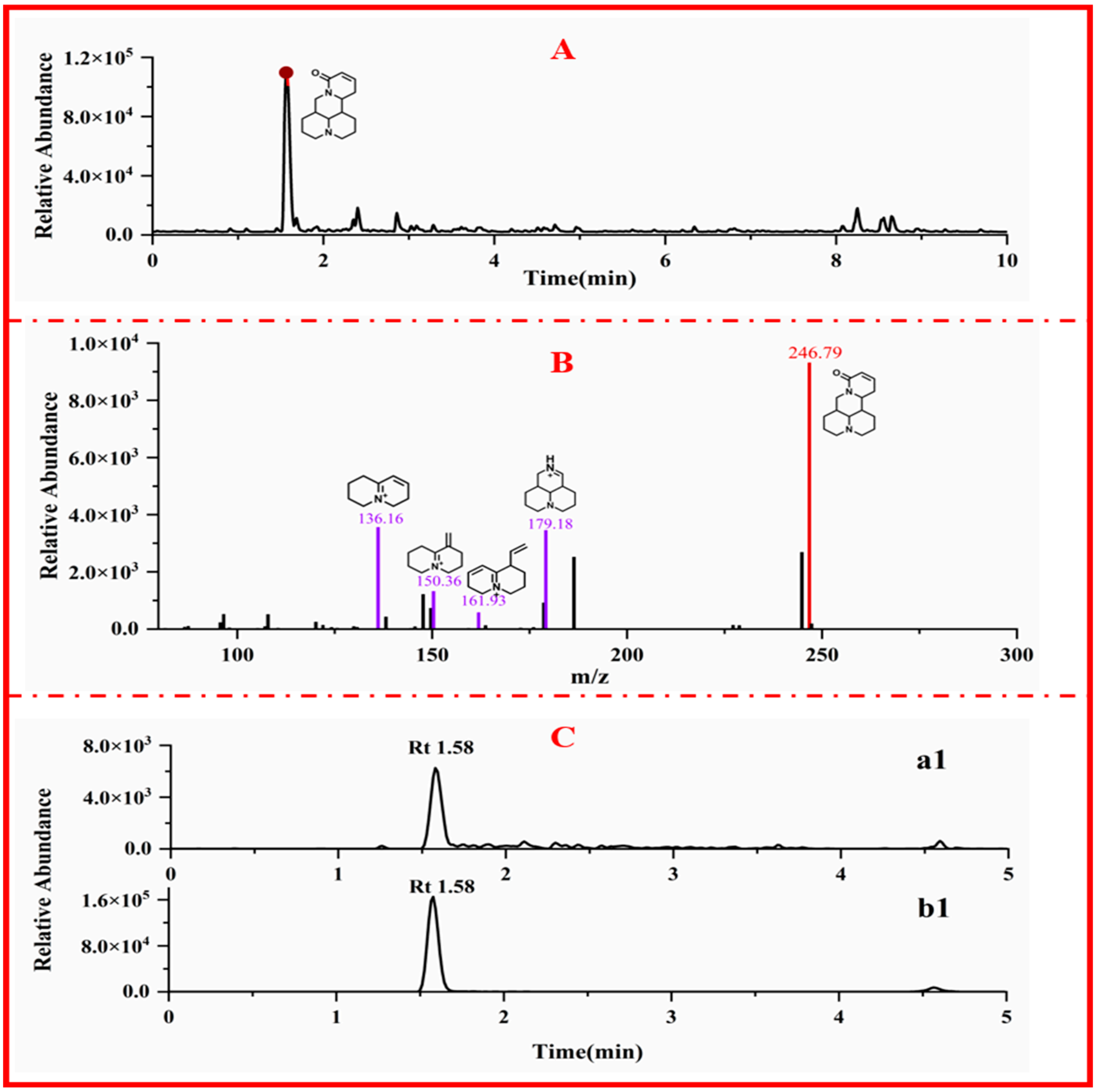

Supplementary Figure F1 EIC diagram and MS/MS spectrum of M3. A is EIC diagram of M3, B is MS/MS spectrum of M3 (The mass-to-charge ratio and structure of each fragment of the compound M3 are labeled on this diagram of B); C are EIC diagrams of sample and sophocarpine standard reference, where a1 is sample and b1 is standard reference.

The molecular formula of metabolite M4 was determined to be $C_{15}H_{22}N_2O_2$ (m/z 263.17 $[M+H]^+$), which exhibited a mass 16Da more than that of M3 (m/z 247.17 $[M+H]^+$). Notably, the major MS/MS fragment ions of M4 were observed at 245.67, 150.15, 148.36 and 136.35, which closely resemble the fragment ions of oxysophocarpine. As shown in **Supplementary Figure F2**, M4 and oxysophocarpine have the same retention time (1.58min).

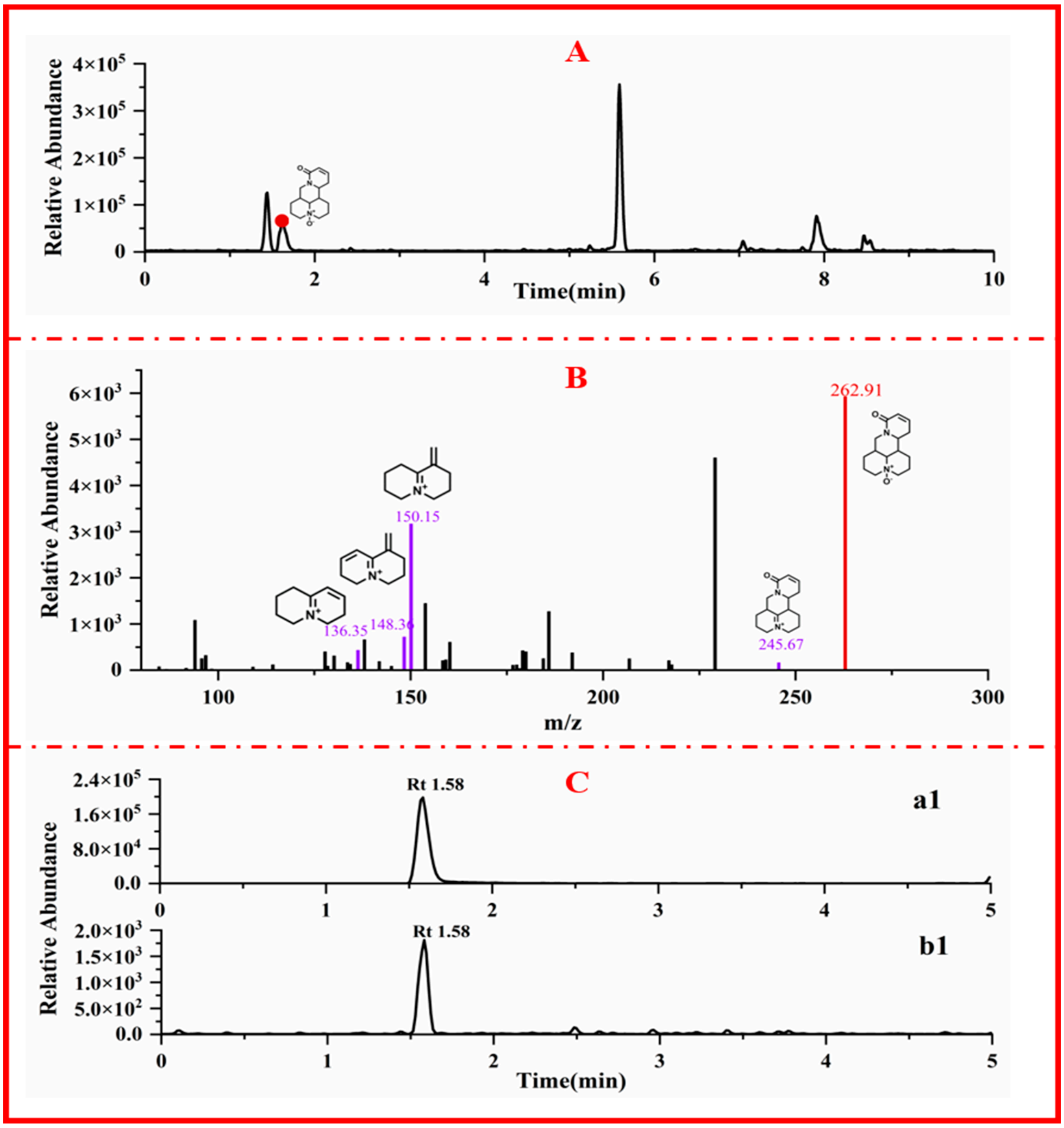

Supplementary Figure F2 EIC diagram and MS/MS spectrum of M4. A is EIC diagram of M4, B is MS/MS spectrum of M4 (The mass-to-charge ratio and structure of each fragment of the compound M4 are labeled on this diagram of B); C are EIC diagrams of sample and oxysophocarpine standard reference, where a1 is sample and b1 is standard reference.

The molecular formula of metabolite M5 was determined to be $C_{15}H_{24}N_2O_2$ (m/z 265.18 $[M+H]^+$), which exhibited a mass 2Da more than that of M4 (m/z 263.17 $[M+H]^+$). It was suspected that M5 may be a reduction product of M4. As shown in **Supplementary Figure F3**, the major MS/MS fragment ions of M5 were observed at m/z 150.00, 148.04, 136.17 and 122.28, which closely resemble the fragment ions of M1. There is also speculation that M5 is an oxidative metabolite of M1 based on the presence of characteristic fragment ions in the MS/MS spectrum. After verification by the oxymatrine standard reference, the two compounds have the same retention time.

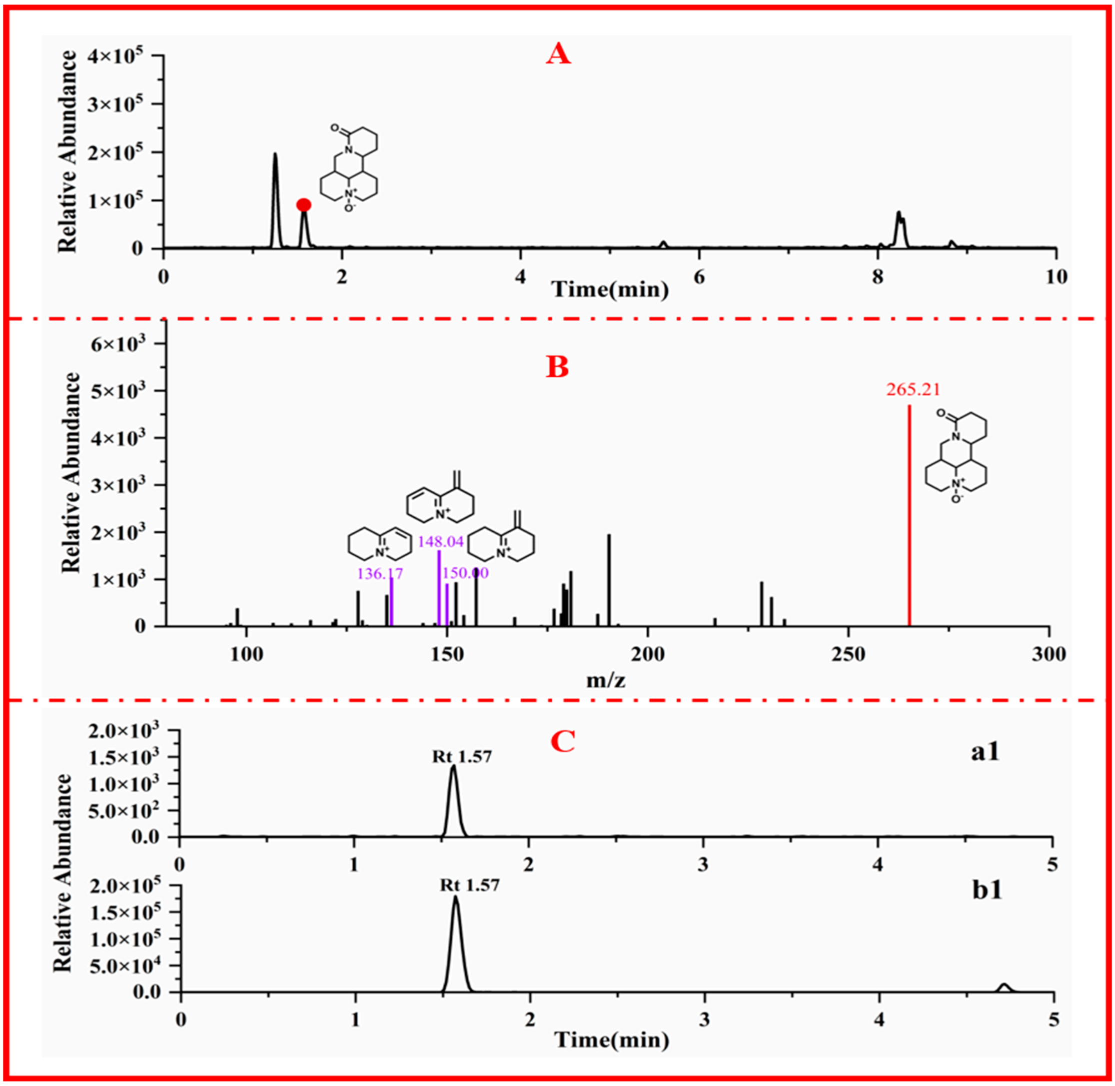

Supplementary Figure F3 EIC diagram and MS/MS spectrum of M5. A is EIC diagram of M5, B is MS/MS spectrum of M5 (The mass-to-charge ratio and structure of each fragment of the compound M5 are labeled on this diagram of B); C are EIC diagrams of sample and oxymatrine standard reference, where a1 is sample and b1 is standard reference.

The molecular formula of metabolite M6 was determined to be $C_{16}H_{29}N_3S$ (m/z 296.21 $[M+H]^+$), which exhibited a mass 2Da more than that of MASM (m/z 294.19 $[M+H]^+$). The major MS/MS fragment ions were observed at m/z 263.17, 239.07, 110.05 and 179.05, which closely resemble the fragment ions of MASM. M6 identification was confirmed as a reduction metabolite of MASM using synthetic deuterium-labeled MASM. The EIC, MS/MS spectrum of M8 and EIC, MS/MS spectrum of isotopic markers were all showed in **Supplementary Figure F4**.

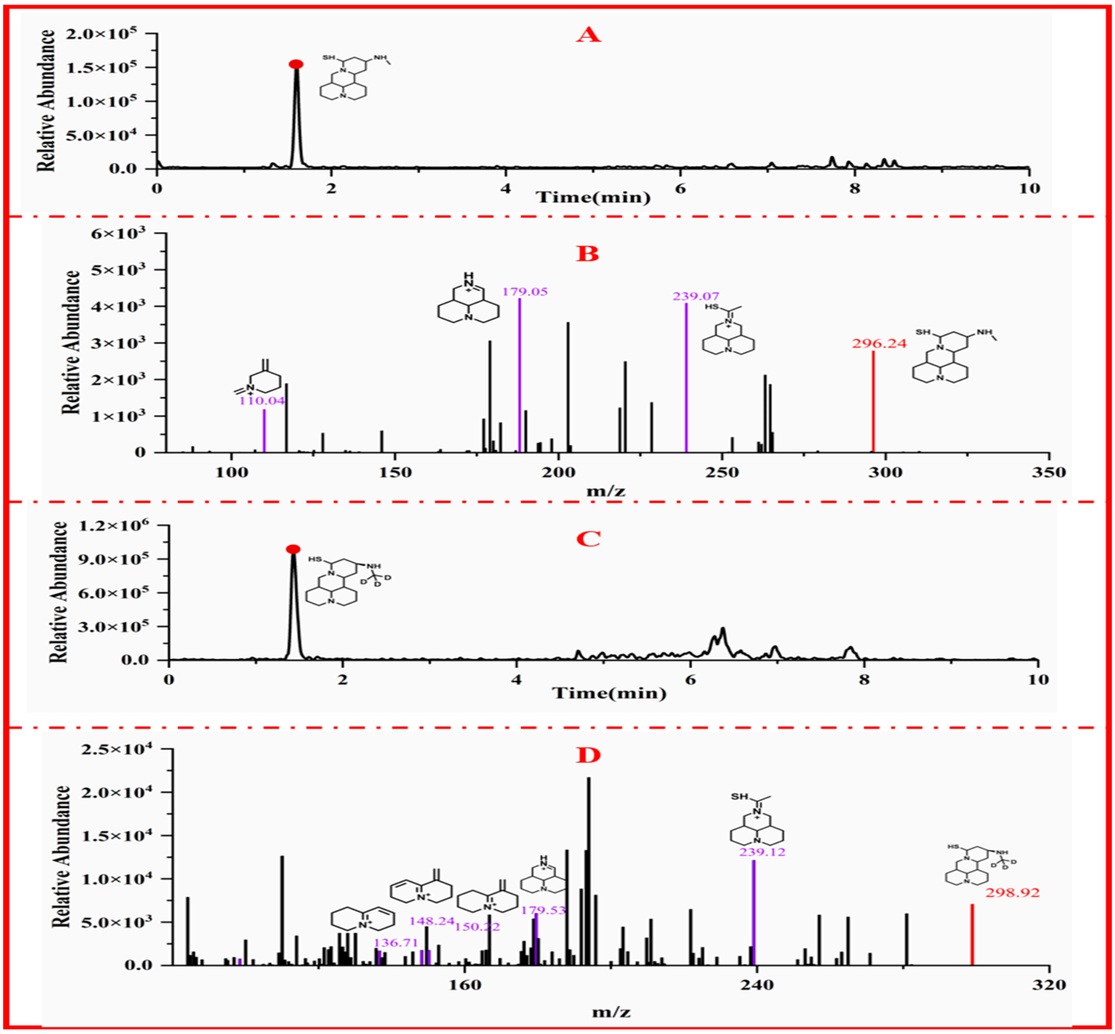

Supplementary Figure F4 EIC diagram and MS/MS spectrum of M6. A is EIC diagram of M6, B is MS/MS spectrum of M6 (The mass-to-charge ratio and structure of each fragment of the compound M6 are labeled on this diagram of B); C is EIC diagram of deuterium-labeled M6; D is MS/MS spectrum of deuterium-labeled M6 (The mass-to-charge ratio and structure of each fragment of the compound deuterium-labeled M6 are labeled on this diagram of D).

The molecular formula of metabolite M7 was determined to be $C_{15}H_{25}N_3S$ (m/z 280.18 $[M+H]^+$), which exhibited a mass 14Da less than that of MASM (m/z 294.19 [M+H]+). The major MS/MS fragment ions were observed at m/z 179.26, 149.85 and 147.75, which closely resemble the fragment ions of MASM. Based on the characteristic fragment ions appeared in the MS/MS spectrum, M7 was identified as a demethyl metabolite of MASM. The EIC diagram, MS/MS spectrum were showed in **Supplementary Figure F5**.

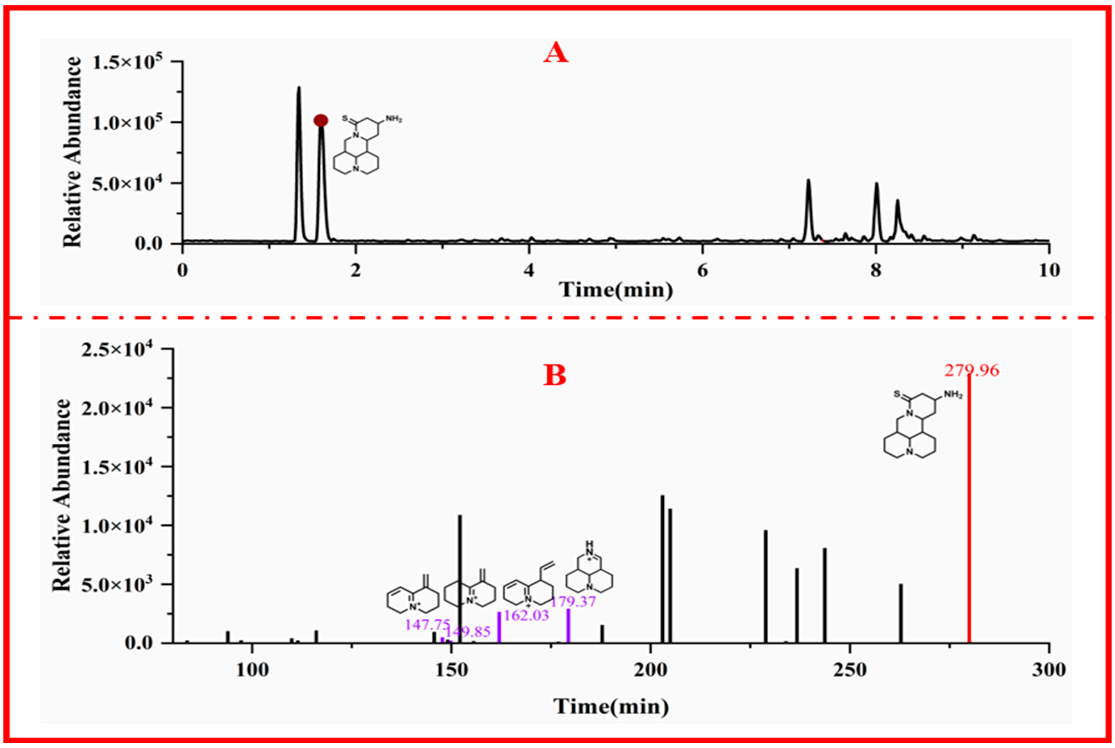

Supplementary Figure F5 EIC diagram and MS/MS spectrum of M7. A is EIC diagram of M7, B is MS/MS spectrum of M7 (The mass-to-charge ratio and structure of each fragment of the compound M7 are labeled on this diagram of B).

The molecular formula of metabolite M9 was determined to be $C_{16}H_{27}N_3OS$ (m/z 310.19 $[M+H]^+$), which exhibited a mass 16Da more than that of MASM (m/z 294.19 $[M+H]^+$). The major MS/MS fragment ions were observed at m/z 110.02, 176.66 and 187.99, which is consistent with the characteristic cleavage law of matrine alkaloid. Based on the characteristic fragment ions appeared in the MS/MS spectrum, M9 was identified as a hydrogenation metabolite of MASM. The EIC diagram and MS/MS spectrum were showed in **Supplementary Figure F6**.

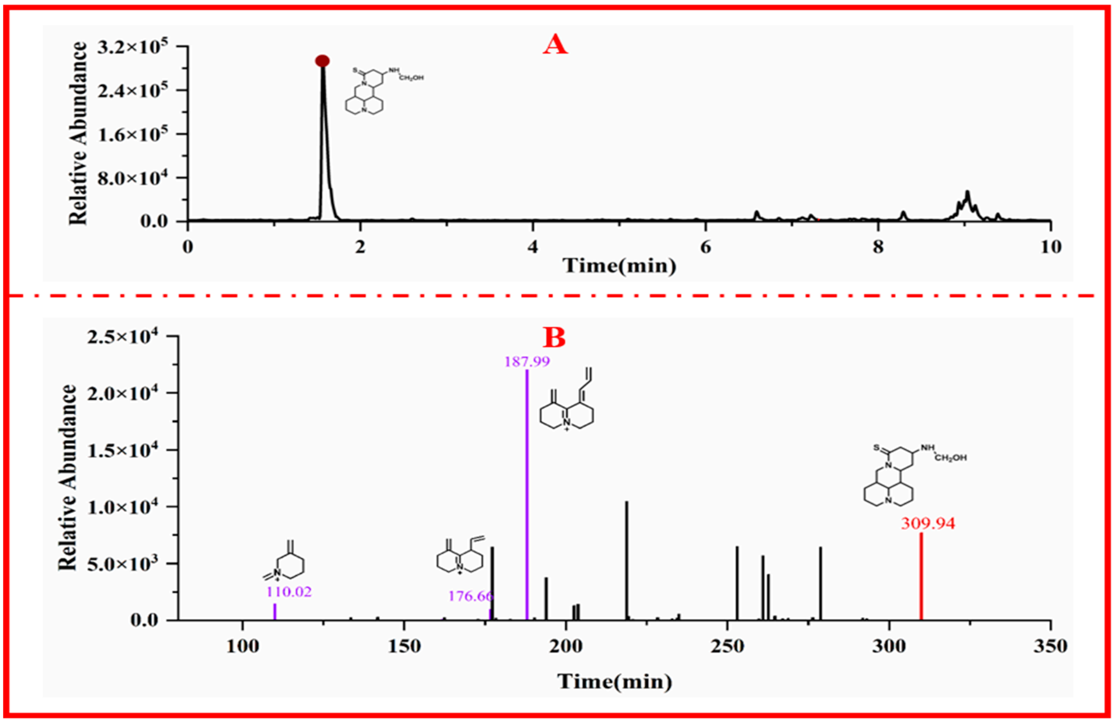

Supplementary Figure F6 EIC diagram and MS/MS spectrum of M9. A is EIC diagram of M9, B is MS/MS spectrum of M9 (The mass-to-charge ratio and structure of each fragment of the compound M9 are labeled on this diagram of B).

The molecular formula of metabolite M10 was determined to be $C_{16}H_{25}N_3OS$ (m/z 308.17 $[M+H]^+$), which exhibited a mass 2Da less than that of M9 (m/z 310.19 $[M+H]^+$). The major MS/MS fragment ions were observed at m/z 122.09, 148.16, 192.93 and 233.05, which is consistent with the characteristic cleavage law of matrine alkaloid. Based on the characteristic fragment ions appeared in the MS/MS spectrum, M10 was identified as an oxidative metabolite of M9. The EIC diagram and MS/MS spectrum were showed in **Supplementary Figure F7**.

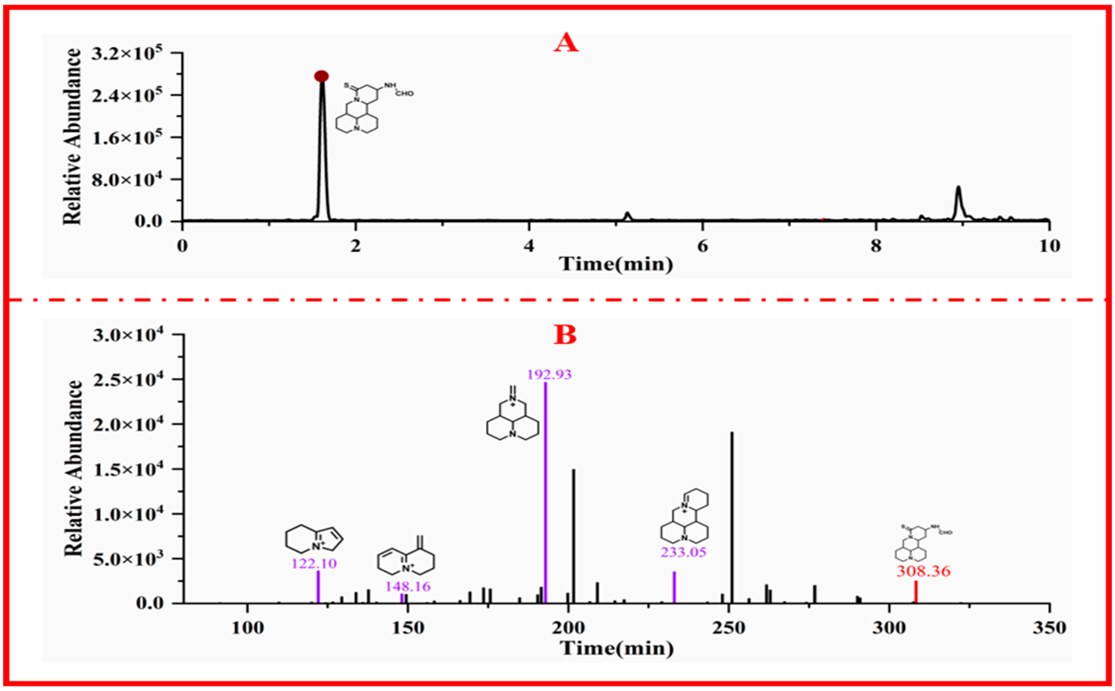

Supplementary Figure F7 EIC diagram and MS/MS spectrum of M10. A is EIC diagram of M10, B is MS/MS spectrum of M10 (The mass-to-charge ratio and structure of each fragment of the compound M3 are labeled on this diagram of B).
